# Database of diffuse reflectance infrared Fourier transform spectroscopy (DRIFTS) and hyperspectral imaging (HSI) spectra of pigments and dyes for historical document analysis

**DOI:** 10.1007/s00216-025-05948-3

**Published:** 2025-06-17

**Authors:** Anna Sofia Reichert, Ana Belén López-Baldomero, Francisco Moronta-Montero, Ana López-Montes, Eva María Valero, Carolina Cardell

**Affiliations:** 1 Department of Painting, Faculty of Fine Arts, University of Granada, Av. Andalucía s/n, 18071 Granada, Spain; 2 Department of Optics, Faculty of Sciences, University of Granada, Campus Fuentenueva, s/n, 18071 Granada, Spain; 3 Department of Mineralogy and Petrology, Faculty of Sciences, University of Granada, Campus Fuentenueva, s/n, 18071 Granada, Spain

**Keywords:** Spectral library, Painting mock-ups, Diffuse reflectance Fourier transform infrared spectroscopy (DRIFTS), Hyperspectral Imaging (HSI), Illuminated and decorated manuscripts, Non-contact analyses

## Abstract

Characterizing pigments and dyes in historical manuscripts is challenging due to the fragility of materials, the complex composition of low-concentration elements, and sampling limitations. Consequently, complementary non-invasive analytical techniques and non-contact measurement methods are often required. This study presents the most comprehensive spectral database to date, combining diffuse reflectance infrared Fourier transform spectroscopy (DRIFTS) and hyperspectral imaging (HSI) to aid in identifying pigments, dyes, and binders historically used in illuminated and decorated manuscripts. A total of 156 painting mock-ups were created using traditional techniques, incorporating variations in binders, pigment particle sizes, support types, surface roughness, and application methods. Spectral imaging was performed in the visible and near infrared (VNIR) and short-wave infrared (SWIR) regions, while DRIFTS analysis covered the middle wave infrared (MWIR) region. For DRIFTS, both contact and non-contact measurements were tested. Using the samples in the database, the influence of binder, support, and grain size on the sample spectra and color were analyzed and discussed. This database facilitates pigment and dye identification using DRIFTS or HSI data independently or in combination through data fusion, applying techniques ranging from direct spectral comparison to advanced methods such as machine learning and spectral unmixing. By making this database publicly available, the study underscores the value of DRIFTS and HSI in identifying painting materials and contributes to the preservation of historical manuscripts.

## Introduction

Characterizing historical illuminated and decorated manuscripts is challenging due to their complex composition and vulnerability to most laboratory-based analytical techniques. The need for conservation has led to a growing number of historical-artistic and analytical studies [1–3], aiming to expand knowledge on painting techniques and materials. Identifying these materials is crucial for selecting compatible treatments and conservation strategies [4, 5]. However, analytical studies remain difficult due to sampling constraints, requiring non-invasive approaches [6] and the refinement of suitable analytical techniques, such as HSI systems [7].

Spectroscopic techniques, including Fourier transform infrared spectroscopy (FTIR), Raman spectroscopy, X-ray fluorescence spectrometry (XRF), and fiber optics reflectance spectroscopy (FORS), have proven effective for identifying dyes and pigments mixed with binding media [4, 8–11]. These techniques provide valuable insights into composition, provenance, and degradation processes [12, 13]. Advances in portable analytical methods have further expanded their use in cultural heritage studies, offering less invasive approaches through non-contact measurements [5], which help preserve the integrity of artifacts [13, 14]. However, fluorescence interference and the high sensitivity of some supports to laser excitation [15] limit the use of Raman spectroscopy to devices that allow strict control of laser radiance [16, 17]. As a result, FTIR, including diffuse reflectance Fourier transform infrared spectroscopy (DRIFTS), is among the most widely used techniques for in situ analyses of historical documents. Its main advantage lies in the simultaneous identification of organic and inorganic materials, though limitations include interferences due to low material quantities, surface roughness, and spectral distortions [18].

Hyperspectral imaging (HSI) has emerged as a powerful tool for acquiring high-resolution reflectance spectra [19]. It provides both spatial and spectral data, enabling precise identification of pigments, dyes, and binders, as well as underdrawings, layered structures, and restorations—especially when combined with automated classification and machine learning methods [20, 21]. However, to prevent heating and bleaching caused by infrared and ultraviolet radiation, proper data acquisition conditions are essential [22]. Additionally, reference materials of known composition are often needed, emphasizing the importance of complementing HSI with elemental or molecular analytical techniques.

A key requirement for identifying painting materials is the development and use of comprehensive spectral databases. These allow material recognition by comparing spectral features with known substances, ideally incorporating multiple variables that influence analytical results. While numerous databases exist for painting materials [10, 13, 23–27], most focus on specific characteristics, such as material type (organic [27], inorganic [25]), origin (natural [27], synthetic [26]), color [4], or composition [11]. Few include spectra acquired using multiple analytical techniques. Additionally, most studies emphasize oil and fresco techniques [28], whereas tempera-based mixtures remain relatively underexplored [10, 29], highlighting the need for more extensive data [30] and further research on these materials.

Historical manuscripts present unique challenges due to their fragility, the thinness of paint layers with high binder content, and the influence of the support on spectral data. Therefore, it is essential to use databases with specifically designed painting mock-ups that replicate materials commonly used in illuminated and decorated manuscripts. This study introduces a combined DRIFTS and HSI database based on more than 150 mock-ups of traditional dyes and pigments applied to paper and parchment, with up to quadriphasic mixtures. The database includes DRIFTS spectra in the mid-infrared (MWIR) region (4000–650 cm⁻^1^) and HSI spectra in the visible- and near infrared (VIS, 400–1000 nm) and short-wave infrared (SWIR, 900–1700 nm) ranges. Additional information regarding pigment composition and particle size was obtained through X-ray diffraction (XRD), micro-Raman (µ-Raman), and laser granulometry analyses. This work highlights the complexity of identifying mixed materials in illuminated and decorated manuscripts and details the process of creating an extensive spectral database. While this work presents the study of manuscripts as a main focus, it also holds significant value for research in multiple other fields due to the inclusion of pure pigment spectra, which can be compared with a wide range of polychrome artworks. The data is stored in widely used open-license formats to ensure accessibility and usability for future research. The characterization results contribute to understanding pigment/dye-binder interactions and evaluating the advantages and limitations of combining two non-invasive analytical techniques.

## Materials and methods

### Pigments, dyes, binders, and supports

All painting materials used in this study are historically documented in illuminated and decorated manuscripts. The mock-ups were created following traditional painting techniques [1, 9]. Egg glair and gum Arabic were selected as binders due to their historical prevalence in the Western world [31]. Pigments, dyes, and gum Arabic were sourced from Kremer Pigmente GmbH & Co. KG. The selected supports included handmade paper and parchment. The paper, composed of a 1:1 mixture of cotton and linen fibers, was obtained from Paperlan® (Gijón, Spain), while the parchment was acquired from Roemer Shop® (Galuburg, Germany). To replicate historical preparation methods, the parchment underwent a “degreasing” process [32], in which calcium carbonate (CaCO₃) was homogeneously applied with a linen cloth, and the excess was removed using a soft brush.

Eighteen pigments and five dyes were obtained in powder form and characterized using laser granulometry, X-ray diffraction (XRD), micro-Raman (µ-Raman), and DRIFTS, following the methodology described in “Analytical techniques.” These pigments and dyes were then mixed with either egg glair or gum Arabic. Table 1 provides their commercial names, chemical compositions, and particle sizes as specified by the supplier. The painting materials are grouped by color, and a list of the abbreviated names used in this study is also included.

**Table 1:** Characteristics of pigments and dyes according to manufacturer (*Kremer Pigmente®*)

Kremer® reference code		Author’s reference	Kremer® chemical description	Kremer® particle size (µm)
Pigments				
10207	Azurite MP, Sky-Blue Light	AZ-EF	Azurite Cu_3_(CO_3_)_2_(OH)_2_	< 38
10206	Azurite MP, Light	AZ-M	Azurite Cu_3_(CO_3_)_2_(OH)_2_	38—63
10204	Azurite MP, Dark	AZ-C	Azurite Cu_3_(CO_3_)_2_(OH)_2_	63—80
10203	Azurite MP, Extra dark	AZ-EC	Azurite Cu_3_(CO_3_)_2_(OH)_2_	80—100
10010	Smalt, very fine	SM	Blue glass, Co-silicate	< 80
10562	Lapis Lazuli from Chile	LAP	(Na,Ca)_8_(Al,SiO_4_)_6_(S,SO_4_, Cl)_x_	38—45
46000	Cremnitz White*	LW	PbCO_3_	n.i
58720	Calcite	CA-EF	Calcite CaCO_3_	20
10624	Cinnabar, very fine	CIN	Cinnabar HgS	< 20
48651	Hematite, intense tinting	HMT	Hematite Fe_2_O_3_	1—10
42500	Red Lead, Minium** ~ **	MIN	Minium Pb_3_O_4_	< 63
10700	Orpiment, genuine	ORP	Orpiment As_4_S_6_	175
10110	Lead Tin Yellow Deep (type I)	LTY	Lead stannate Pb_2_SnO_4_	< 38
10300	Malachite natural, standard	MLC	Malachite Cu_2_(CO_3_)(OH)_2_	< 120
44450	Verdigris, synthetic	VG	Copper(II)-acetate-1-hydrate Cu(CH_3_COO)_2_·[Cu(OH)_2_]_3_·2H_2_O	n.i
116421	Yellow Moroccan Ochre, fine	OC	Pure earth pigment from central Morocco	< 80
40710	Burnt Umber, brownish	BU	Natural brown earth, contains manganese oxides	n.i
12015	Grape Seed Black	GB	Charred Grape Seeds	n.i
Dyes				
42100	Carmine Naccarat	CARM	Aluminium lake of carminic acid, C_22_H_2_OO_13_	n.i
37050	Gamboge, powder	GMB	Gamboge (H_3_0_1_) 100%	n.i
37110	Saffron, red threads	SA	Crocus sativus	n.i
36000	Indigo, genuine	IND	Natural organic product. Natural Blue 1, C.I. 75780. Indigosfera species or Isatis tinctoria	n.i
37380	Ripe buckthorn berries	BCKT	Natural Yellow 13	n.i

*Cremnitz white is an equivalent to lead white (LW). ~ Red lead is the synthetic pigment and minium (MIN) the natural pigment. *n.i.* not identified

### Painting mock-ups

A total of 156 mock-ups were created using the previously mentioned painting materials, combining up to four components bound with either gum Arabic or egg glair (see Table 2 for details on the mixtures included in the database). Several additional factors were evaluated to assess their influence on the analytical results, including the type of support, the type of binder, pigment particle size (specifically for azurite), and method of paint application (either as a mixture or in superimposed layers).

As a result, the database includes three monophasic samples (consisting of binders or liquid dyes that do not require a binder), 39 biphasic mixtures (one pigment/dye + one binder), 34 triphasic mixtures (two pigments/dyes or their combinations + one binder), and two quadriphasic mixtures (three pigments + one binder). Each formulation was applied to both paper and parchment. For triphasic and quadriphasic mock-ups, the pigment-to-binder ratios used in biphasic mixtures were maintained, following 1:1 and 1:1:1 proportions, respectively. Since these ratios are not standardized, quantities were adjusted based on two factors: the maximum pigment concentration admitted by the binder—known as Critical Pigment Volume Concentration (CPVC)—and the desired fluidity of the paint.

Gum Arabic (GA) was prepared at a 20% concentration in water (20:100 w/v), while egg glair was obtained following traditional methods by beating the egg white, removing the surface foam, and adding water to achieve a 1:1 volume ratio. Imperial yellow ink was prepared according to the Kremer® recipe, which involves adding potash alum and gum Arabic to a buckthorn solution.

**Fig. 1:**
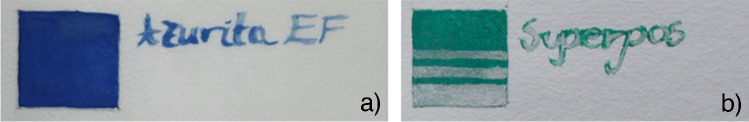
Examples of painting mock-ups bound with gum Arabic: **a** azurite extra fine on parchment and **b** superimposition of lead white and malachite applied on paper

The prepared paints were applied to paper and parchment in 2 × 2 cm squares and as written text using a brush (Fig. 1a). To evaluate variations induced by different application methods, pigments were either mixed homogeneously and applied in a single layer or applied as separate superimposed layers using the same components (Fig. 1b).

Table 2 shows the taxidermy of the database samples according to the number and proportion of painting materials present. The exact composition of the mock-ups, including total binder content, can be found in the general information of the database (*Information_Mock-ups*).

**Table 2:** Characteristics of the painting mock-ups: number and type of components and their proportions in the paint mixtures. Paints are applied either in uniform mixtures or in superimposed layers on paper and parchment supports

Type of mock-up	Pigments and dyes	Binders	Proportion	Total N^o^. of mock-ups
*Monophasic*	-	Gum Arabic (GA)	20:100 (w/v)	6
	-	Egg glair (EG)	50:50 (v/v)	
	Ripe buckthorn berries * (BCKT)	Pure	0,3:1 (w/v*)*	
*Biphasic*	Azurite EF (AZ-EF)	Gum Arabic or egg glair	1:0,5 (w/v)	78
	Azurite M (AZ-M)	Gum Arabic or egg glair	1:0,5 (w/v)	
	Azurite C (AZ-C)	Gum Arabic or egg glair	1:0,5 (w/v)	
	Azurite EC (AZ-EC)	Gum Arabic or egg glair	1:0,5 (w/v)	
	Smalt (SM)	Gum Arabic or Egg glair	1:0,5 (w/v)	
	Lapislazuli (LAP)	Gum Arabic or egg glair	0,4:1 (w/v)	
	Lead White (LW)	Gum Arabic or egg glair	0,4:1 (w/v)	
	Calcite (CA)	Gum Arabic or egg glair	0,4:1 (w/v)	
	Cinnabar (CIN)	Gum Arabic or egg glair	0,4:1 (w/v)	
	Hematite (HMT)	Gum Arabic or egg glair	0,4:1 (w/v)	
	Minium (MIN)	Gum Arabic or egg glair	0,2:1 (w/v)	
	Orpiment (ORP)	Gum Arabic or egg glair	0,33:1 (w/v)	
	Lead Tin Yellow (LTY)	Gum Arabic or egg glair	0,5:1 (w/v)	
	Malachite (MLC)	Gum Arabic or egg glair	2:1 (w/v)	
	Verdigris (VG)	Gum Arabic or egg glair	2:1 (w/v)	
	Yellow Ochre (OC)	Gum Arabic or egg glair	0,2:1 (w/v)	
	Burnt Umber (BU)	Gum Arabic or egg glair	0,4:1 (w/v)	
	Grape Seed Black (GB)	Gum Arabic	0, 4:1 (w/v)	
	Carmine* (CARM)	Gum Arabic	2:1 (w/v)	
	Gamboge* (GMB)	Gum Arabic	2:1 (w/v)	
	Indigo* (IND)	Gum Arabic	0,66:1 (w/v)	
	Saffron* (SA)	Gum Arabic	2:1 (v/v)	
*Triphasic*	AZ-EF + LW ^ϴ^	Gum Arabic or egg glair	1:1:1 (v/v/v)	68
	LTY + LW ^ϴ^	Gum Arabic or Egg glair	1:1:1 (v/v/v)	
	MLC + LW ^ϴ^	Gum Arabic or Egg glair	1:1:1 (v/v/v)	
	VG + LW ^ϴ^	Gum Arabic or egg glair	1:1:1 (v/v/v)	
	CIN + LW ^ϴ^	Gum Arabic or egg glair	1:1:1 (v/v/v)	
	GB + LW ^ϴ^	Gum Arabic or egg glair	1:1:1 (v/v/v)	
	MIN + LTY	Gum Arabic or egg glair	1:1:1 (v/v/v)	
	AZ-EF + GMB	Gum Arabic	1:1:1 (v/v/v)	
	MLC + GMB	Gum Arabic	1:1:1 (v/v/v)	
	VG + GMB	Gum Arabic	1:1:1 (v/v/v)	
	ORP + IND	Gum Arabic	1:1:1 (v/v/v)	
	GMB + SA	Gum Arabic	1:1:1 (v/v/v)	
	CARM + GMB	Gum Arabic	1:1:1 (v/v/v)	
	LW + CARM	Gum Arabic	1:1:1 (v/v/v)	
	Imperial Yellow Ink (buckthorn + alum)	Gum Arabic	0,18:0,01:1 (w/w/v)	
*Quadriphasic*	LW + CIN + LTY	Gum Arabic or egg glair	1:1:1:1 (v/v/v/v)	4

* = Dye. ^ϴ^ = Samples made using two types of paint application (in homogeneous mixtures and in layers). See Table 1 to check the painting materials’ name abbreviations. Weight = w (g); volume = v (ml). Pure saffron was prepared with 7,5 saffron threads in water, which is equivalent to a 0,0124:25 (w/v) proportion

### Analytical techniques

#### Laser granulometry (LG)

Particle size measurements were conducted using a Mastersizer 2000LF equipped with a Hydro 2000G accessory (Malvern Instruments®). Since ethanol was used as the dispersing medium, the refractive index was manually adjusted to *n* = 1.36. For each sample, three measurements of 30 s were taken, and the average values were calculated. Results were analyzed based on the volume distribution of particle sizes, assuming a uniform sample distribution [33], and are presented in Table 3.

#### X-ray diffraction (XRD)

Powder X-ray diffraction (XRD) was performed using a PANalytical X’PertPRO diffractometer with Cu-Kα radiation and silicon zero-background sample holders. The instrument operated at 45 kV and 40 mA, with an exploration range of 3 to 60° 2θ and a goniometer speed of 0.01° 2θ/s. In samples where Powder XRD results were inconclusive (i.e., yellow ochre and burnt umber), µ-XRD analyses were conducted using a Bruker D8 DISCOVER diffractometer equipped with a DECTRIS PILATUS3R 11 K-A detector and a 1 mm diameter X-ray beam. The operating conditions included Cu-Kα radiation, 50 kV voltage, 1 mA intensity, an exploration range of 10 to 57° 2θ, and a scanning speed of 0.02° with 40 s per step. Crystalline phase identification was carried out using *Xpert Highscore 2.0* and *Profex 5.2.8*, both linked to the Crystallography Open Database. Results are presented in Table 3.

#### Micro-Raman (μ-Raman)

μ-Raman analysis was performed only in specific cases where XRD results lacked sufficient resolution, such as for black carbon-based pigments (i.e., grape seed black). Measurements were conducted using a confocal JASCO NRS-5100 Micro-Raman Spectrometer, coupled with a Peltier-cooled CCD detector and an Olympus microscope. The working conditions included a spectral range of 300–2000 cm⁻^1^ with a resolution of 1 cm⁻^1^. Samples were excited using two lasers: a green laser (Elforlight G4-30; Nd:YAG; 532 nm) and a red laser (Torsana Starbright; 785 nm). The results of these measurements are presented in Table 3.

#### Diffuse Reflectance Infrared Fourier Transform Spectroscopy (DRIFTS)

DRIFTS analyses were conducted on both painting mock-ups and pigments and dyes in powder form using a portable 4300 Handheld FTIR Spectrometer (Agilent Technologies®) with a Diffuse Reflectance interface and a 6 mm spot diameter. IR spectra were collected in the mid-infrared region (spectral range, 4000 to 650 cm⁻^1^) with 10 scans per spectrum and a resolution of 4 cm⁻^1^. A Coarse Gold Reference Cap (G8180-67560) was used for background subtraction. Spectra were processed with baseline corrections to avoid errors caused by instrument distortions [18]. Data was acquired using both direct contact and non-contact methods with the surface of the mock-ups. Note that only spectra from non-contact measurements are presented, as results from both methods were similar, and non-invasive analysis is preferred in the study of historical documents. To ensure adequate results, the obtained contact and non-contact spectra were compared with the “Cultural Heritage Open Source” (CHSOS) DRIFTS spectral library [6]. The spectra of non-bound pigments closely matched those reported in the library, with minor variations attributable to baseline corrections. These variations, however, do not impact the qualitative interpretation of the results. Furthermore, although the CHSOS database provides DRIFTS spectra exclusively for pigments bound with an acrylic medium, the combined influence of the support and binders was found to be consistent with the signals obtained from our mock-ups, thereby validating the comparative framework.

**Fig. 2:**
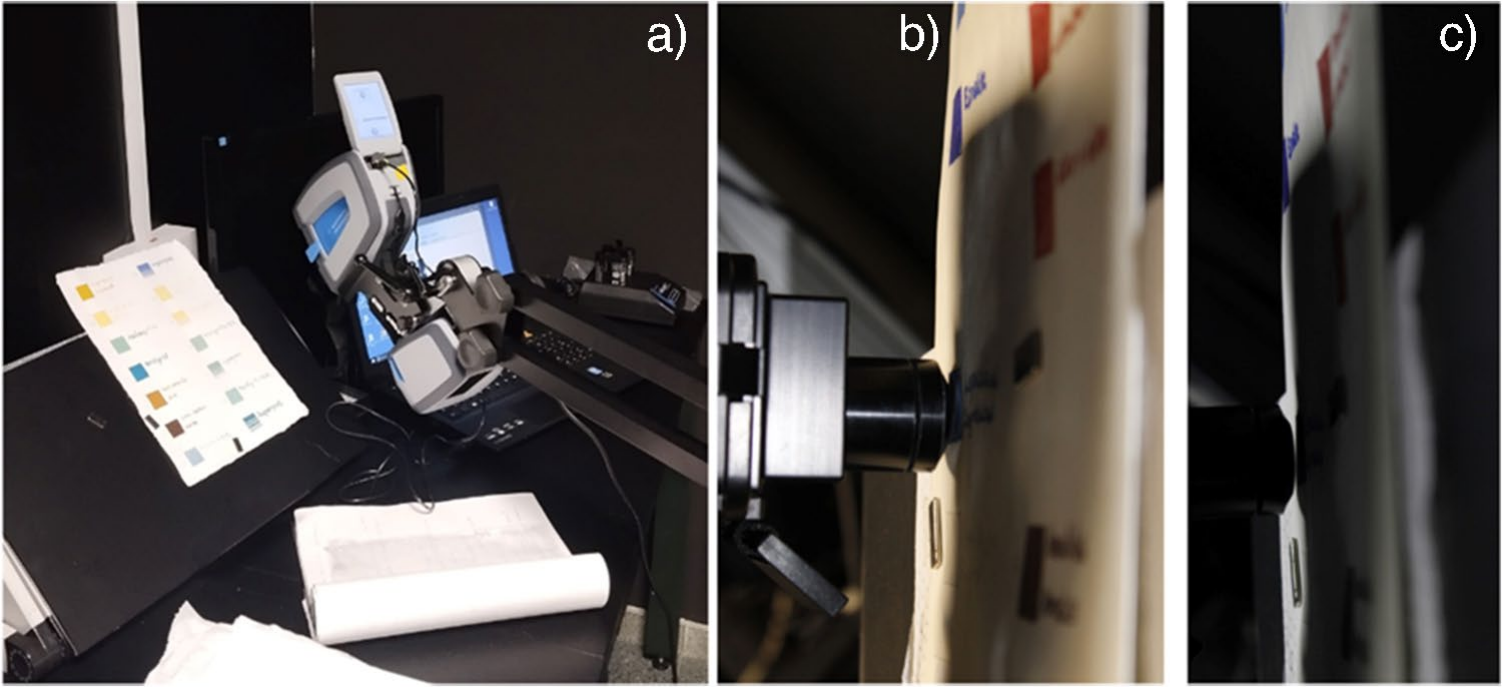
Measurement acquisition system for portable DRIFTS. **a** Instrument placement on the adjustable tripod. **b** and **c** Non-contact analyses with minimal distance to the document surface

To prevent damage to the mock-ups and simulate real data acquisition procedures for historical documents, a custom support system was designed. This system, consisting of an adjustable tripod and a reclining bookrest, allowed analyzing the mock-ups at a minimal distance and under stable conditions for the instrument (Fig. 2).

#### Hyperspectral Imaging (HSI)

HSI measurements were performed with two line-scan spectral imaging cameras from Resonon® Ltd (Pika L and Pika IR +) which allowed acquiring data in two spectral ranges (VNIR and SWIR respectively). The VNIR camera (Pika L) covers the spectral range from 380 to 1080 nm using 900 pixels per line and a spectral resolution of 2.1 nm. The SWIR camera (Pika IR +) covers from 888 to 1732 nm with 640 pixels per line and a 2.4 nm resolution. The outer portions of the range were cropped due to low signal-to-noise ratio, and spectra were interpolated in both spectral ranges with a sampling interval of 5 nm, resulting in spectra containing 121 bands in VNIR (from 400 to 1000 nm) and 161 bands in SWIR (from 900 to 1700 nm). Dark subtraction and flat field corrections were applied, using the 90% reflectance patch from the Sphere Optics Zenith Lite Multistep. The light source was a set of four halogen lamps oriented to avoid specular reflection from the samples. The capture distance was 50 cm for the VNIR range and 40 cm for the SWIR range, with a field of view of 14.5 cm approximately for both cameras, resulting in a spatial resolution of 0.16 mm/pixel for the VNIR and 0.22 mm/pixel for the SWIR camera.

**Fig. 3:**
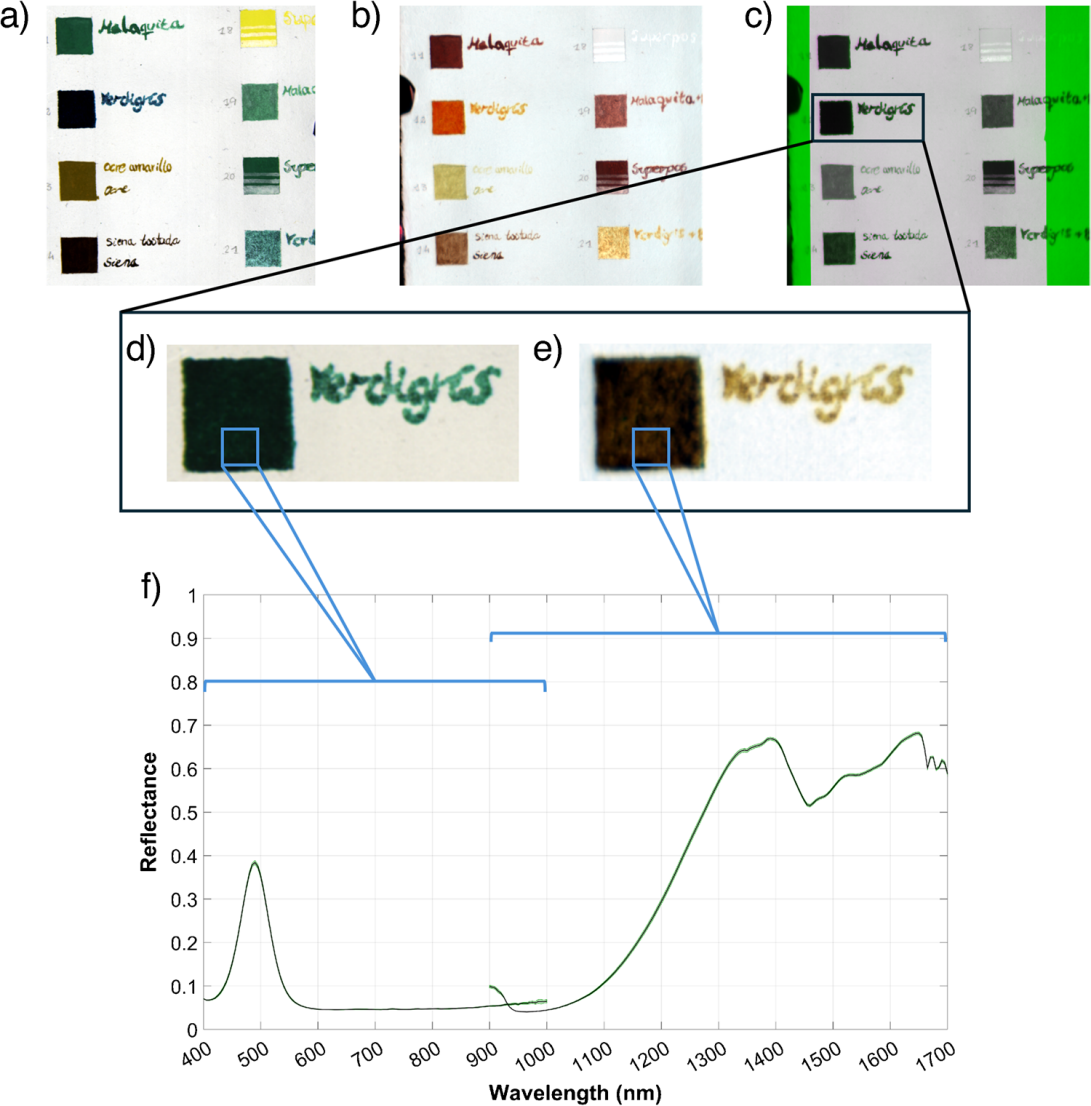
Extraction process of the spectra from verdigris samples. **a**, **b** False color (RGB) images of a portion of the sheets containing the samples in the VNIR and SWIR ranges. The bands used for the false color images are [605, 535, 430] nm and [1600, 1200, 1000] nm, respectively. **c** Overlay of the two bands used for spatial registration of the VNIR and SWIR spectral cubes. **d**, **e** False color images of the verdigris sample cropped from the registered images in the VNIR and SWIR ranges, respectively. **f** Concatenated average reflectance spectrum with standard deviation

The samples were captured in groups corresponding to the different sheets of either paper or parchment support on which they were deposited. To ensure a spatial correspondence between VNIR and SWIR sample cubes, the spectral images of the VNIR and SWIR sheets were registered using the Registration Estimator App in MATLAB, as shown in Fig. 3c. Afterwards, a region of interest (ROI) of 20 × 20 pixels was extracted from the painted square in each sample and each spectral range in the registered cubes. The ROI size was chosen to cover 19.4 mm^2^, approximately 70% of the spot area of the handheld FTIR device. The spectral reflectance curves of the 400 pixels in the ROI were averaged. The averaged spectral data was stored in CSV format as part of the database, including the two spectral ranges (first VNIR and then SWIR). Some variations can be found in the overlapping region (900–1000 nm) resulting from the use of two different imaging devices. This occurs for several reasons, such as differences in the spectral bandwidth and the fact that these wavelengths correspond to the extremes of both sensors’ ranges, where sensitivity decreases.

In addition to the averaged spectra with standard deviation plots included in the database (accessible at Supplementary Information), the ROI hyperspectral images with pixel-by-pixel reflectance are available in BIL and H5 formats. This constitutes one of the unique traits of this database, since it allows direct examination of the spatial uniformity of the samples. Figure 3 illustrates the process of extraction of the spectra from one of the samples, including the average and standard deviation spectral reflectance plot.

#### Color analysis

Color coordinates were extracted from HSI reflectance data and calculated pixelwise using CIE D65 standard illuminant and the 2-degree CIE 1931 standard colorimetric observer. Mean values for each sample were calculated by averaging the color coordinates of the pixels within the ROI in CIELAB and CIEL*C*h* color spaces. In the CIELAB space, L* represents clarity (ranging from black with a value of 0 to white with a value of 100), and a* and b* represent the axis red (+ a*) to green (− a*) and yellow (+ b*) to blue (− b*). CIEL*C*h* cylindrical coordinates correspond to clarity (L*), chroma (C*_ab_), and hue (h*_ab_). To study the influence of binders, supports, and azurite particle size, ΔE*_ab_ color differences were calculated using the CIE 1976 formula (ΔE*_ab_ = √(ΔL*)^2^ + (Δa*)^2^ + (Δb*)^2^). The results are presented in “Color analysis.” Besides, color differences using the CIEDE2000 color difference formula [34, 35] were calculated and are accessible through the Supplementary Information.

## Results and discussion

### XRD and laser granulometry

The mineralogical composition and particle size of the pigments studied here, some of which were analyzed in previous works [36–40], are shown in Table 3. The first observation confirms that the supplier’s specifications for commercialized pigments are not always accurate and often include additional materials [36]. This factor must be considered in scientific studies and conservation or restoration treatments and requires the use of combined analytical techniques to ensure reliable results [41, 42].

The results highlight the presence of impurities, such as quartz, identified in azurite, calcite, cinnabar, hematite, and malachite pigments in previous studies [35], and in yellow ochre and burnt umber pigments in this study. These impurities are generally not indicated by the manufacturer. Additionally, dolomite (detected in hematite [35]), calcite, and kaolinite (detected in yellow ochre and burnt umber, respectively) are identified, and their presence can typically be attributed to the natural origin of the pigments.

Therefore, characterizing the mineralogy and particle size of the pigments is essential to avoid incorrect interpretations of results obtained from DRIFTS and HSI analysis. The mineralogical composition and particle size of dyes were not studied due to their organic nature. μ-Raman analysis was exclusively applied to characterize the grape seed black pigment, which is primarily composed of carbon.

**Table 3:** Mineralogical composition and particle size of pigments studied in this work. Particle size ranges are presented between brackets, preceded by main maximum particle size

Kremer® reference	Author’s reference	Chemical composition	Particle size (µm)
Azurite MP, 10207	AZ-EF	Azurite, malachite, quartz *	25 (4–90) *
Azurite MP, 10206	AZ-M	Azurite, malachite, quartz *	45 (20–110) *
Azurite MP, 10204	AZ-C	Azurite, malachite, quartz *	70 (25–180) *
Azurite MP, 10203	AZ-EC	Azurite, malachite, quartz *	90 (20–280) *
Smalt, 10010	SM	n.d	55 (1–100) †
Lapis Lazuli, 10562	LAP	Lazurite, calcite, diopside †	47 (0,6–95) †
Cremnitz White 46000	LW	Hidrocerussite, cerussite •	3 (0,1–10) •
Calcite, 58720	CA-EF	Calcite, dolomite, quartz ~	25 (0,25–100) ~
Cinnabar, 10624	CIN	Cinnabar#	12 (0,4–40) •
Hematite, 48651	HMT	Hematite, quartz, dolomite #	0,6 (0,3–17) #
Red Lead, 42500	MIN	Minium #	3 (0,4–9) #
Orpiment, 10700	ORP	Orpiment	42 (0,2–84)
Lead Tin Yellow, 10110	LTY	Hidrocerussite, cassiterite	3,2 (0,2–15)
Malachite, 10300	MLC	Malachite, pseudomalachite •	3 (0,2–112) †•
Verdigris, 44450	VG	Hoganite, tenorite	102,5 (1,8–226)
Yellow Ochre, 116421	OC	Goethite, quartz, kaolinite	10,5 (0,2–76)
Burnt Umber, 40710	BU	Hematite, quartz, calcite	6,4 (0,2–42)
Grape Seed Black, 12015	GB	Calcite, carbon	84 (0,3–152)

Information according to: * = Cardell et al. (2017) [37]; † = Pozo-Antonio et al. (2020) [38]; • = Pozo-Antonio et al. (2022) [39]; ~  = Rivas et al. 2018 [40]; # = Pozo-Antonio et al. (2018) [36]. *n.d.* not detected using XRD. Quartz, SiO_2_; lazurite, Na_6_Ca_2_(Al_6_Si_6_O_24_)(SO_4_,S,S_2_,S_3_,Cl,OH)^2−^; diopside, MgCaSi_2_O_6_; hidrocerussite, Pb_3_(CO_3_)_2_(OH)_2_; cerussite, PbCO_3_; dolomite, CaMg(CO_3_)_2_; cassiterite, SnO_2_; pseudomalachite, Cu_5_(PO_4_)_2_(OH)_4_; hoganite, Cu(CH_3_COO)_2_H_2_O; tenorite, CuO; goethite, α-Fe^3+^O(OH); kaolinite, Al_2_Si_2_O_5_(OH)_4_; carbon, C. See Table 1 for the chemical composition of the rest of the painting materials

### DRIFTS and HSI

**Fig. 4:**
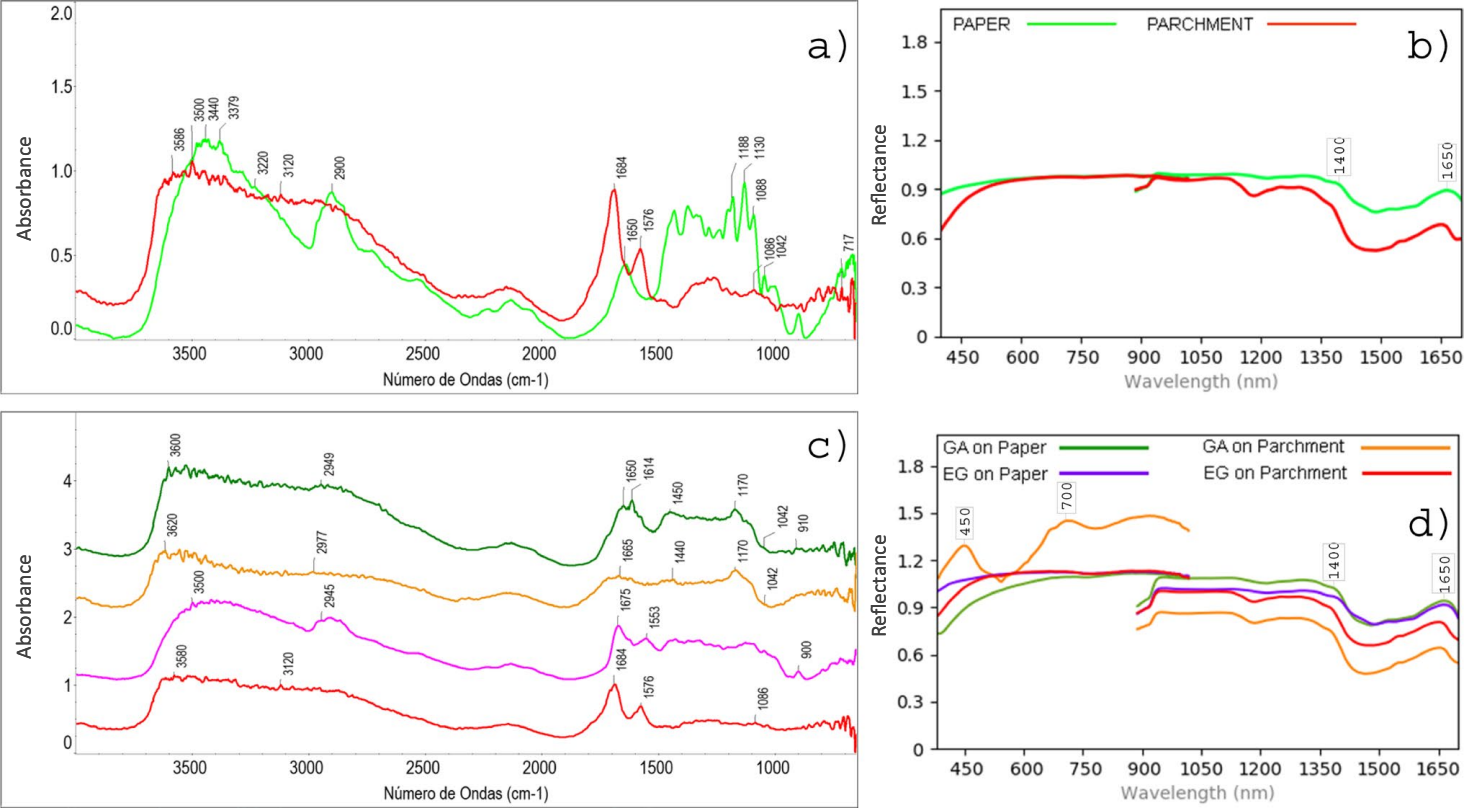
DRIFTS and HSI spectra of supports and binders. **a**, **b** Support’s DRIFTS and HSI spectra. **c**, **d** Binder mock-up’s DRIFTS and HSI spectra, respectively. See Table 1 to consult names abbreviations. Readers are referred to the online version of this article for better interpretation of spectra

**Fig. 5:**
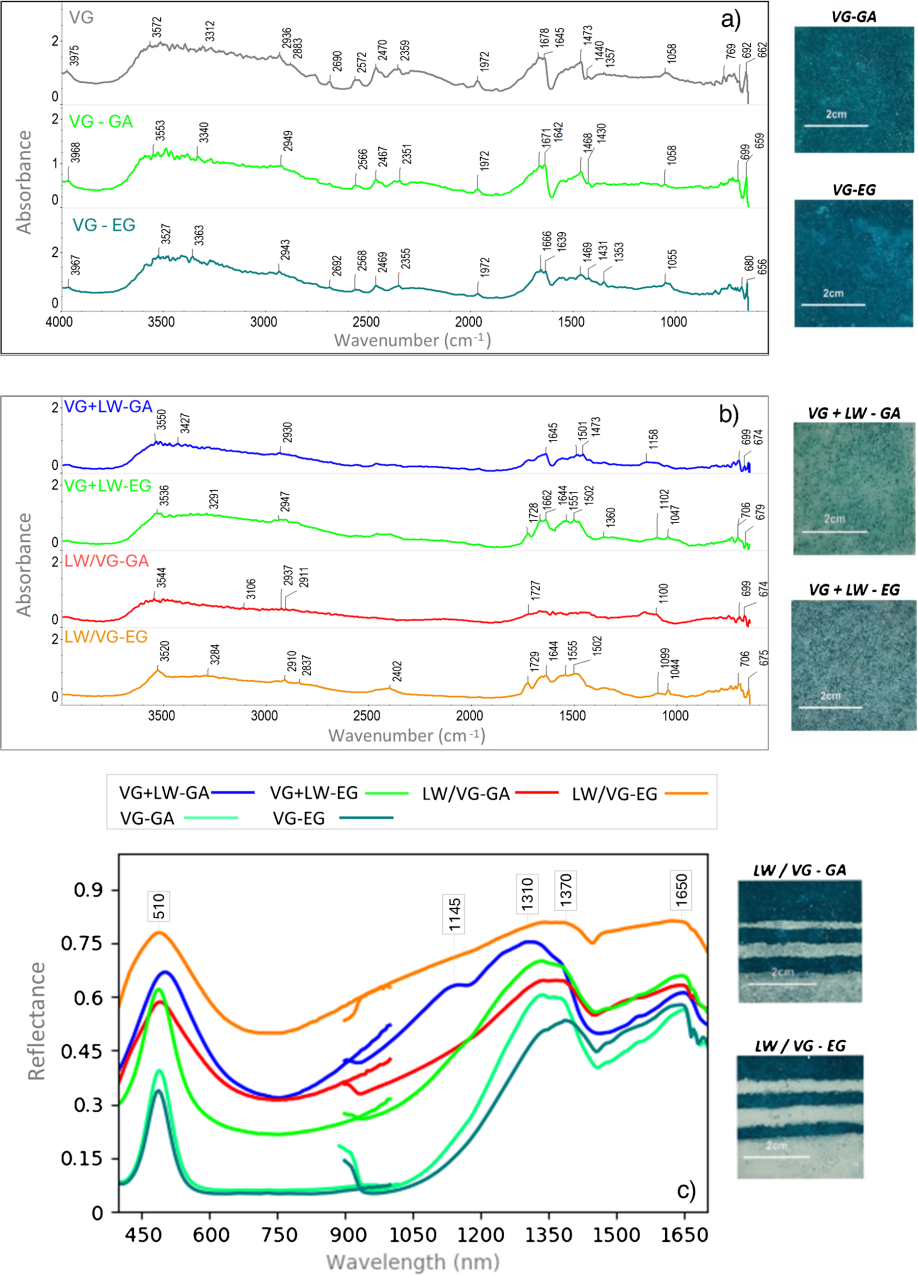
DRIFTS spectra of **a** pure verdigris and its biphasic mixtures, **b** triphasic mixtures with different types of paint application (+ = uniform mixture;/= superimposed layers), and **c** HSI reflectance spectra of biphasic and triphasic mixtures, all of which are placed on parchment. See Table 1 for names abbreviations

**Fig. 6:**
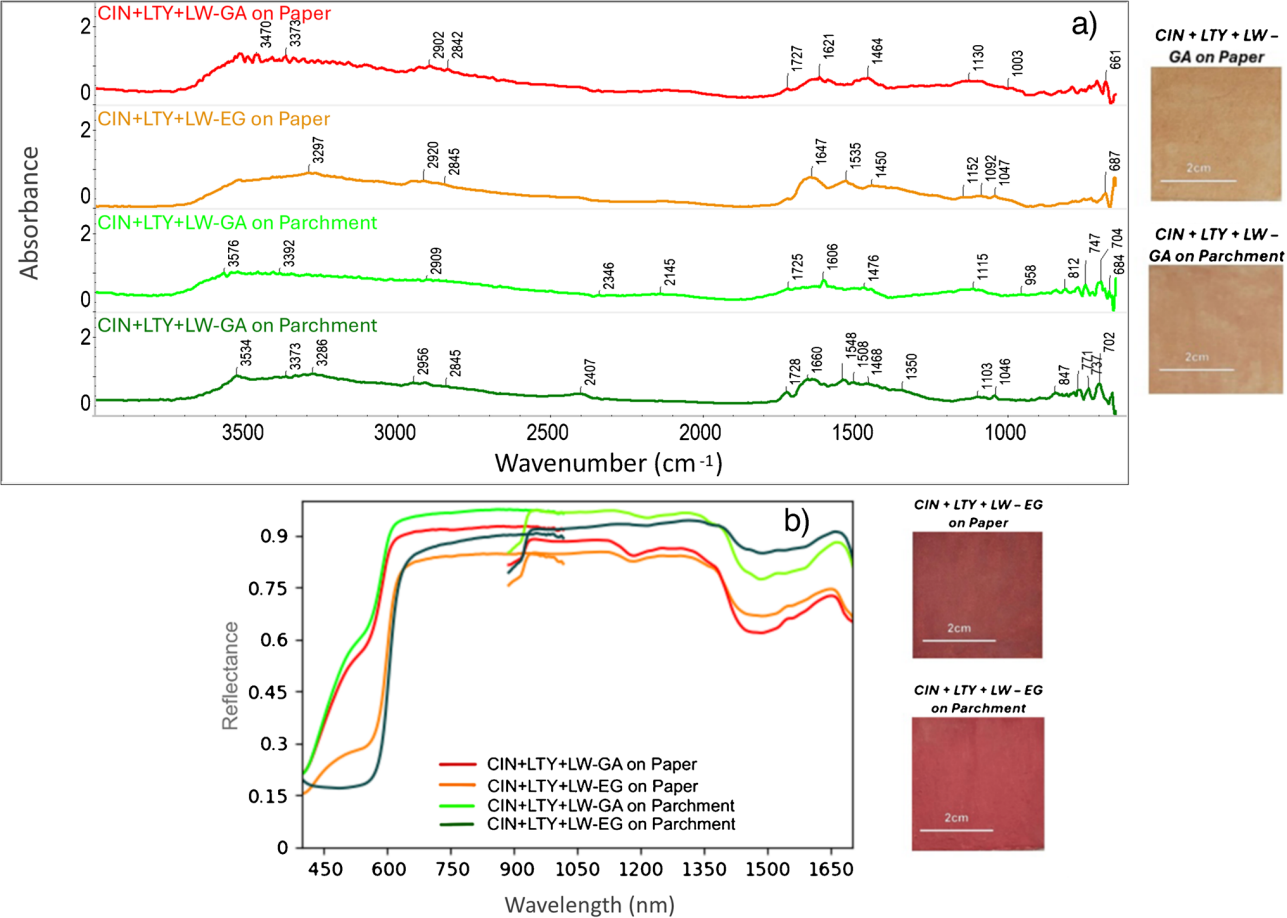
**a** DRIFTS and **b** HSI spectra of the quadriphasic mixture (cinnabar (CIN) + lead tin yellow (LTY) + lead white (LW)) bound with GA (gum Arabic) or EG (egg glair) on paper and parchment supports

This section presents the combined DRIFTS and HSI spectra of supports (i.e., paper and parchment) and binders (i.e., gum Arabic and egg glair) (Fig. 4), followed by examples of spectra of verdigris painting mock-ups of varying complexity (monophasic, biphasic, and triphasic) (Fig. 5) and one quadriphasic mixture (Fig. 6). As mentioned in the materials and methods section (“Painting mock-ups”), the type of paint application was also considered, and it was found to have a considerable impact on the results (Fig. 5). Finally, the influence of particle size and pigment/dye-binder interactions in mixed paintings was evaluated only for azurite mock-ups.

The combined contribution of these variables to the DRIFTS and HSI spectra is one of the strengths of this database, as it provides comparative results on the influence of variables commonly encountered in historical documents (e.g., different supports, binders, and particle sizes). Since this paper primarily focuses on the study of mock-ups, spectra for pure pigments can be accessed in the Supplementary Information, as is the usual practice in previous databases [6]. An example of pure verdigris pigment is, however, included in Fig. 5 as a reference to evaluate the spectral changes induced by painting mixtures (biphasic and triphasic mixtures) applied on parchment with different binders and types of paint application.

#### Supports and binders

Figure 4 presents the DRIFTS and HSI spectra of the analyzed supports (paper and parchment) and binders (gum Arabic -GA- and egg glair -EG-). The organic nature of both binders in DRIFTS analyses was confirmed by the presence of CH functional groups (spectral ranges 3400–3300 cm^−1^ (alkynes), 3100–3000 cm (alkenes), and 3000–2800 cm^−1^ (alkanes). Cellulose bands of the paper support (Fig. 4a) correspond to OH (3600; 3200–3100 cm^−1^) and CO (1300–1000 cm^−1^, with main bands at 1042 and 1088 cm^−1^) chemical bonds. Bands at 1650 cm^−1^ are attributed to OH stretching (*v*) and bending (*β*,*δ*) vibrations of water molecules [43]. Parchment (Fig. 4a) shows characteristic bands of CH (organic), NH (protein), and OH (water) functional groups in the regions from 3600 to 2800 cm^−1^. Its proteinaceous nature is confirmed by two sharp bands at 1684 cm^−1^ (amides -C = O-) [44] and at 1576 cm^−1^ (NH stretching) [45]. The band at 1086 cm^−1^ (CO_3_^2−^ (*v*_*sym*_)) was associated with calcite (CaCO_3_) [11] used in the degreasing process. Furthermore, when evaluating DRIFTS spectra, bands between 2400 and 2300 cm^−1^ attributed to atmospheric CO_2_ [46, 47] must be considered in all obtained results.

Gum Arabic (Fig. 4c) is a polysaccharide compound with main absorption bands between 3700 and 3100 cm^−1^ [48, 49] (CH and OH bonds) and at 2949 cm^−1^ [14]. Bands around 1170 cm^−1^, attributed to CO stretching (1300–1000 cm^−1^), appear more pronounced on paper supports since characteristic absorptions occur in the same region. Note that bands between 1700 and 1400 cm^−1^ (due to OH bending, CH bending, C = O, NH [14], and water bonds) can be masked by interferences from the support, particularly when placed on parchment. The absence of intense bands around 1743 and 1240 cm^−1^, which are attributed to different gums used as binders in artworks (such as tragacanth gum), has been identified as an indicator of gum Arabic [49] and can aid in its characterization.

The spectrum of egg glair mock-ups (Fig. 4c) resembles that of the parchment support (Fig. 4a), due to the similarities in their composition, which result in the presence of bands in the same ranges. This can lead to misleading results. However, when placed on paper, characteristic bands near 2978 cm^−1^ (CH stretching) [14] and 1553 cm^−1^ (NH bending) are more distinguishable.

Regarding the HSI results, the support’s influence mainly translates into an increase in reflectance values on paper and a decrease on parchment due to its darker tone (Fig. 4b), especially in the SWIR range (900–1700 nm). This trend is consistent in binders, with gum Arabic achieving higher reflectance (Fig. 4d). In the VNIR range (400–1000 nm), and particularly between 700–900 nm, binders and supports show similar reflectance values, except for the gum Arabic on parchment mock-up, which exhibits fluctuating reflectance results. This fluctuation is due to the specular reflection of gum Arabic, which was difficult to avoid during hyperspectral captures.

#### Painting mock-ups

This section presents DRIFTS and HSI spectra of selected pigments mixed with either gum Arabic or egg glair, as a representative example of the content of the database. Figure 5 displays the DRIFTS spectra of verdigris (VG) mock-ups on parchment, including their corresponding variations for both binders (Fig. 5a). It also includes biphasic (VG-GA, VG-EG) and triphasic mixtures (VG + LW), as well as the type of paint application (either as uniform mixtures or superimposed layers) (Fig. 5b). The DRIFTS spectra are shown alongside those of the pure verdigris pigment, facilitating the comparison and evaluation of the variations induced by the binder and supports. HSI spectra for the biphasic and triphasic uniform mixtures are presented in Fig. 5c, without standard deviation to improve visualization.

Verdigris pigments show characteristic bands between 1620 and 1400 cm⁻^1^ [10, 45] and 700 and 600 cm⁻^1^, which are associated with the symmetric and asymmetric stretching of acetate groups (-COO- (v_sym_, v_asym_)) [50, 51]. While characteristic bands around 690 cm⁻^1^ are present in all bound mock-ups, position shifts of up to 13 cm⁻^1^ were observed for different supports: 699 cm⁻^1^ (VG-GA on parchment), 681 cm⁻^1^ (VG-GA on paper), 680 cm⁻^1^ (VG-EG on parchment), and 677 cm⁻^1^ (VG-EG on paper). However, bands between 1690 and 1550 cm⁻^1^ (NH (v,*β and δ*)), which are attributed to proteinaceous compounds like egg glair and parchment, present significant overlaps with those of the pure verdigris pigment. Additionally, co-occurrences with the characteristic band of water at 1640 cm⁻^1^ [28] must be considered to avoid erroneous interpretations.

As shown in Fig. 5a, mock-ups bound with egg glair exhibit a higher absorbance intensity compared to those bound with gum Arabic. The broader bands between 3400 and 2800 cm⁻^1^ are due to organic CH bonds and, therefore, cannot be attributed solely to the pigment, supports, or binders, as all of these materials present at least partially organic compositions. Furthermore, Fig. 5b shows the DRIFTS spectra for mixtures of verdigris and lead white. The presence of lead white is indicated by bands around 1100 cm⁻^1^, which are attributed to symmetric stretching of carbonates (CO₃^2^⁻). Characteristic bands of cerussite, typically located around 1735, due to CO_3_^2−^ symmetric stretching [44], and at 2400 cm⁻^1^ [14], were measured in pure pigments (accessible through Supplementary Information) but could not be identified correctly in these mock-ups due to the combined masking effects of verdigris, binders, and supports. The attribution of OH stretching molecular vibrations to hydrocerussite at 3800–3200 cm^−1^ [14, 47] is equally unclear given the previous statement. The type of paint application is mainly distinguished by a decrease in the intensity of the characteristic verdigris bands in mock-ups obtained through superimposed layers, as the upper layer corresponds to lead white, which exhibits greater absorbance.

Regarding HSI results, Fig. 5c indicates reflectance variations and, consequently, color changes in biphasic and triphasic mixtures of verdigris (VG) and lead white (LW) in uniform mixtures. The results show that reflectance is higher when lead white is included as a component. Interestingly, triphasic mock-ups bound with gum Arabic exhibit higher reflectance values in the VNIR range, while reflectance for both biphasic and triphasic mock-ups bound with egg glair is higher in the SWIR range, mainly from 1400 to 1700 nm. This trend is generally maintained, as observed in Fig. 6b, suggesting that the type of binder can potentially be detected depending on the considered spectral range.

To present a more complex example, Fig. 6 shows DRIFTS and HSI spectra of the quadriphasic mixture included in the database (cinnabar (CIN) + lead tin yellow (LTY) + lead white (LW)). Bands from 1552 to 1160 cm⁻^1^ are attributed to sulfides (S^2−^) found in cinnabar, although presenting a slight shift when compared to the characteristic band referenced by Manfredi et al. (2017) [14], which should be located at 1129 cm⁻^1^. Additionally, bands around 1250 cm^−1^ have been attributed to SO_2_ stretching [47], similarly to those described at 1233 cm^−1^ for orpiment pigments (arsenic trisulfide (As_2_S_3_)) in previous studies [14]. Finally, bands between 1100 and 700 cm⁻^1^ suggest the presence of Si–O bonds proceeding from quartz [47, 52] while those around 1450–1420, 1100–1000, and 720–700 cm^−1^ (CO_3_^−2^) are associated with calcite [53], both previously identified minerals in CIN pigments by XRD (Table 3). On the other hand, identifying the LTY pigment in the mixture is challenging, as its characteristic bands are located in regions below 650 cm⁻^1^, which is the limit of the spectral measurement range in the portable DRIFTS instrument used. Nonetheless, bands at 1049 and 1040 cm⁻^1^ have been associated with this pigment, as well as with litharge at 679 cm⁻^1^ in previous literature [14]. Bands at 757 and 759 cm^−1^ have been described for Pb–O bonds in lead-based pigments but are generally not clearly resolved [52]. Finally, the presence of LW is detected through bands around 1100 cm⁻^1^ (CO₃^2^⁻), as well as through bands at 2400 [14], 1735, and 840 cm⁻^1^, attributed to the combination of symmetric stretching (*v*_*sym*_) and in-plane bending (*β*) of cerussite (PbCO_3_) [14, 54]. Bands close to 790 cm^−1^ have been identified as β(Pb-OH) bonds and likely correspond to hydrocerussite (Pb_3_(CO_3_)_2_(OH)_2_ [47]. Once more, limitations caused by the spectral range must be considered. The presence of acute bands at 1606 cm⁻^1^ (Fig. 6a), attributed to vibrations of water molecules from the environment [28], is most accentuated in the quadriphasic mixture and must be taken into consideration as an influential factor when using non-contact analyses.

HSI reflectance spectra for this mixture (CIN + LTY + LW) show the influence of the binders (Fig. 6b), with higher values obtained by gum Arabic in the VNIR range and by egg glair in the SWIR range, confirming the findings in Fig. 5. The characteristic relative position of the bands in both spectral ranges is maintained, demonstrating the high value of HSI for material identification in artworks and the importance of having reference samples available for comparison.

Table 4 presents the precise DRIFTS band assignments and HSI reflectance maxima for the mock-ups discussed in the paper, selected as references to illustrate the obtained data and provide a clearer understanding of the results. As shown, maximum reflectance is similar for painting mixtures made with the same pigment but applied to different supports, although standard deviations of up to approximately 60 nm should be considered. Cinnabar mock-ups exhibit the most significant DRIFTS band shifts due to the influence of the supports. These interferences have previously been highlighted as a limitation in DRIFTS analysis by Tamburini et al. [55]. A complete list of DRIFTS band assignments can be accessed in the Supplementary Information.

**Table 4:** DRIFTS absorption bands and suggested assignments, together with HSI reflectance maxima of representative mock-ups and related mixtures

Mock-up	Support	DRIFTS wavenumber (cm^−1^)	DRIFTS attribution	HSI reflectance maxima (nm)
**CIN + GA**	Paper	*3760*^1^, *3569*^1^, *3475*^*2*^, *3420*^*2*^, *3125*^2^, *2929*^2^, *2128*^2^, *1602*^1,9^, ***1410***^**3**,4,5^, ***1371***^*3,4*^, *1293*^5^, ***1242***^4,5,6^, ***1152***^5,7,10^, ***980***^*5*,7,9,10^, **886**^8,9^, **779**^8,10^, ***718***^*3*,10^, **669**^10^	^1^OH(*v*);^2^CH(*v*);^3^CH(*δ,β);*^4^CO_3_^2−^; ^5^CO(*v*);^6^SO_2_(*V*_*asym*_);^7^SO_2_;^8^CO_3_^2−^(*β*); ^9^OH(*δ,β);*^*10*^Si-O	630, 990, 1140, 1660
	Parchment	3517^1^, 3462^1,3^, *3190*^2^, *2928*^2^, *2880*^2^, *2671*^*2,10*^, *2119*^2^, *1635*^1,11^, *1612*^4^, ***1534***^4,6^, *1406*^*5,6,7*^, ***1364***^5,6^, *1318*^5,6^, ***1246***^6,7,8^, ***1153***^7,9,12^, ***978***^7,9,12^,, **833**^10,11^, **774**^5,12^, **661**^12^	^1^OH(*v*);^2^CH(*v*);^3^NH(v);^4^NH(*δ,β);* ^5^CH(*δ,β);*^6^CO_3_^2−^;^7^CO(*v*);^8^SO_2_(*V*_*asym*_); ^9^SO_2_;^10^CO_3_^2−^(*β*); ^11^OH(*δ, β);* ^12^Si-O	630, 930, 1100, 1300, 1660
**CIN + EG**	Paper	*3718*^1^, *3421*^1,3^, *3314*^2,3^, *3075*^2^, *2953*^2^, *2929*^2^, *2145*^2^, *1646*^1,4,11^, ***1544***^4,6^, *1442*^5,6,7^, *1404*^5,6,7^, ***1309***^5,6^, ***1235***^6,7,8^, ***1073***^7,9,10^, **877**^10,11^, **718**^5,12^, **659**^12^	^1^OH(*v*);^2^CH(*v*);^3^NH(v);^4^NH(*δ,β);* ^5^CH(*δ,β);*^6^CO_3_^2−^;^7^CO(*v*);^8^SO_2_(*V*_*asym*_); ^9^SO_2_;^10^CO_3_^2−^(*β*); ^11^OH(*δ, β);* ^12^Si-O	660, 930, 1100, 1400, 1655
	Parchment	*3785*^1^, 3409^1,3^, *3317*^2,3^, 3291^2,3^, *3081*^2^, *2951*^2^, *2925*^2^, *1639*^1,4,12^, ***1533***^4,6^, *1444*^5,6,7^, ***1311***^5,6^, ***1242***^6,7,8^, ***1160***^7,9^, ***1077***^7,9,10^, **877**^11,12^, **752**^5,13^, **704**^5,13^, **660**^13^	^1^OH(*v*);^2^CH(*v*);^3^NH(v);^4^NH(*δ,β);* ^5^CH(*δ,β);*^6^CO_3_^2−^;^7^CO(*v*);^8^SO_2_(*V*_*asym*_); ^9^SO_2_;^10^CO_3_^2−^(*v*_*sym*_);^11^CO_3_^2−^(*β*); ^12^OH(*δ,β);* ^13^Si-O	660, 940, 1120, 1300, 1655
**LTY + GA**	Paper	*3519*^1^, *3390*^1^, *3341*^2^, *3164*^2^, *2862*^2^, *2120*^*2*^, *1621*^1,8^, ***1426***^**5**,6,7^, **1093**^5,7^, **895**^**6**,7,8^, **812**^**6**,9^, **782**^9^, **720**^**3**,6,9^, **704**^3,6,9^	^1^OH(*v*);^2^CH(*v*);^3^CH(*δ,β);*^4^CO_3_^2−^; ^5^CO(*v*);^6^Pb-O;^7^CO_3_^2−^(*β*);^8^OH(*δ,β);* ^9^Si-O	540, 935, 1130, 1310,1520, 1665
	Parchment	3520^3^, 3482^3^, *3395*^2^, *3346*^2^, *3212*^1^, *2931*^2^, *2128*^*2*^, *1622*^1,4,10^, ***1161***^7^, **816**^8,11^, **719**^5,8,11^, **697**^8,11^	^1^OH(*v*);^2^CH(*v*);^3^NH(v);^4^NH(*δ,β);* ^5^CH(*δ,β);*^6^CO_3_^2−^;^7^CO(*v*);^8^Pb-O; ^9^CO_3_^2−^(*β*);^10^OH(*δ,β);* ^11^Si-O	550, 940, 1120, 1245, 1300, 1535, 1660
**LTY + EG**	Paper	*3719*^1^, *3283*^3^, 3187^2^, *2955*^2^, *2929*^2^, *1639*^1,4,10^, ***1539***^4,6^, **1432**^7,8,9^, ***1310***^5,6^, **1230**^6,7^, **1084**^7,9,11^, **1040**^9,11^, **919**^6,8,11^, **817**^8,11^, **744**^8^, **719**^**5**,8,11^, **698**^5,8,11^, **657**^11^	^1^OH(*v*);^2^CH(*v*);^3^NH(v);^4^NH(*δ,β);* ^5^CH(*δ,β);*^6^CO_3_^2−^;^7^CO(*v*);^8^Pb-O; ^9^CO_3_^2−^(*β*);^10^OH(*δ,β);* ^11^Si-O	555, 980, 1115, 1390, 1670
	Parchment	*3719*^1^, 3547^1^, 3226^3^, *3055*^2^, *2956*^2^, *2930*^2^, *1657*^1,4,10^, ***1533***^4,6^, **1434**^7,8,9^, **1406**^7,8,9^, ***1310***^5,6^, **1241**^6^, **1049**^12^, **926**^6,8,11^, **797**^8,11^, **730**^8^, **712**^5,8,11^, **697**^8,11^, **659**^11^	^1^OH(*v*);^2^CH(*v*);^3^NH(v);^4^NH(*δ,β);* ^5^CH(*δ,β);*^6^CO_3_^2−^;^7^CO(*v*);^8^Pb-O; ^9^CO_3_^2−^(*β*);^10^OH(*δ,β);*^11^Si-O; ^12^CO_3_^2−^(*v*_*sym*_)	565, 940, 1380, 1660
**LW + GA**	Paper	***3528***^1^, *3467*^2^, *3078*^2^, *2923*^2^, *2848*^2^, **2402**^4^, *2133*^2^, ***1726***^6,10^, *1624*^1^, *1605*^1^, *1473*^4,6^, ***1361***^3,4^, ***1147***^,9,10^, **1101**^9,10^, **987**^5,9^, **892**^7^, **723**^6,12^, **691**^6,9,11^, **668**^11^	^1^OH(*v*);^2^CH(*v*);^3^CH(*δ,β);*^4^CO_3_^2−^; ^5^CO(*v*);^6^Pb-O;^7^CO_3_^2−^(*β*);^8^OH(*δ,β);* ^9^Si-O;^10^CO_3_^2−^(*v*_*sym*_);^11^CO_3_^2−^(*δ*); ^12^*β*(Pb-OH)	690, 960, 1140, 1310,1380, 1660
	Parchment	*3544*^1^, *3106*^2^, *2937*^2^, 2911^2^, **2409**^5^, *2134*^2^, **1727**^7,11^, *1636*^1,3,9^, 1550^3,5^, 1524^3,5^, *1483*^5,7^, **1460**^5,7^, ***1358***^4,5^, ***1154***^11^, **1100**^10,11^, **820**^7,8^, **782**^12^, **689**^13^	^1^OH(*v*);^2^CH(*v*);^3^NH(*δ,β);*^4^CH(*δ,β);*^5^CO_3_^2−^;^6^CO(*v*);^7^Pb-O;^8^CO_3_^2−^(*β*); ^9^OH(*δ,β);* ^10^Si-O; ^11^CO_3_^2−^(*v*_*sym*_); ^12^Pb-OH(*β*); ^13^CO_3_^2−^(*δ*)	720, 1120, 1300, 1545, 1650
**LW + EG**	Paper	3520^1^, *3419*^2,3^, *3296*^2,3^, *2954*^2^, *2913*^2^, **2402**^5^, *2144*^2^, **1728**^8,11^, *1647*^*1,3,10*^, *1610*^1,3,9^, *1528*^3,5^, *1504*^5^, ***1354***^4,6^, **1092**^11,12^, **1045**^12^, **958**^7,11^, **835**^8,9^, **747**^13^, **697**^14^	^1^OH(*v*);^2^CH(*v*);^3^NH(v);^4^NH(*δ,β);* ^5^CH(*δ,β);*^6^CO_3_^2−^;^7^CO(*v*);^8^Pb-O; ^9^CO_3_^2−^(*β*);^10^OH(*δ,β);*^11^Si-O; ^12^CO_3_^2−^(*v*_*sym*_);^13^Pb-OH(*β*);^14^CO_3_^2−^(*δ*)	540, 820, 950, 1140, 1300, 1455, 1655
	Parchment	*3806*^1^, 3520^1,3^, 3284^1^, *2910*^2^, *2837*^2^, **2402**^5^, *2049*^5^, **1729**^8,11^, *1663*^1,3,10^, *1637*^*1,10*^, *1526*^3,5^, *1489*^5,7^, ***1351***^4,6^, **1099**^11,12^, **1044**^12^, **832**^8,9^, **797**^9,13^, **728**^5,13^, **701**^14^, **661**^11^	^1^OH(*v*);^2^CH(*v*);^3^NH(v);^4^NH(*δ,β);* ^5^CH(*δ,β);*^6^CO_3_^2−^;^7^CO(*v*);^8^Pb-O; ^9^CO_3_^2−^(*β*);^10^OH(*δ,β);*^11^Si-O; ^12^CO_3_^2−^(*v*_*sym*_);^13^Pb-OH(*β*);^14^CO_3_^2−^(*δ*)	670, 890, 945, 1120, 1300, 1470, 1650
**VG + GA**	Paper	*3592*^1^, *3447*^2^, ***3292***^2^, ***2984***^2^, **2565**^4^, *2465*^5^, *2242*^2^, **1970**^4^, *1663*^1,9^, ***1642***^1,6,9^, ***1464***^7^, *1350*^3,4^, 1174^10,11^, **1057**^11^, **954**^6,10^, **787**^6,8^, **742**^6^, **708**^6^, **681**^6^	^1^OH(*v*);^2^CH(*v*);^3^CH(*δ,β);*^4^CO_3_^2−^; ^5^CO(*v*);^6^COO(*v*_*sym*_);^7^COO;^8^CO_3_^2−^(*β*); ^9^OH(*δ,β);* ^10^Si-O; ^11^(CO₃^2^⁻)(*v*_*sym*_)	490, 1390, 1520, 1650,1670, 1690
	Parchment	3615^1^, *3397*^2,3^, **1972**^6^, **1802**^6^, *1642*^1,4,8,11^, ***1616***^1,4,8^, ***1542***^9^, ***1431***^10^, *1321*^5,6^, *1152*^12,13^, 1104^12,13^, **951**^8,12^, 814^14^, **711**^8,14^**, 699**^8^	^1^OH(*v*);^2^CH(*v*);^3^NH(v);^4^NH(*δ,β);* ^5^CH(*δ,β);*^6^CO_3_^2−^;^7^CO(*v*);^8^COO-(*v*_*sym*_); ^9^COO-(*v*_*asym*_);^10^COO;^11^OH(*δ,β);* ^12^Si-O;^13^(CO₃^2^⁻)(*v*_*sym*_); ^14^CO_3_^2−^ (*β*)	495, 1330, 1380, 1540,1650, 1690
**VG + EG**	Paper	*3567*^1^, *3489*^3^, 3248^2^, ***2987***^2^, **2550**^6^, *2463*^7^, *2287*^2^, **1968**^6^, **1879**^6^, *1670*^1,4,11^, ***1559***^4,9^, **1464**^10^, **1429**^10^, **1062**^13^, **777**^8,14^, **704**^8^, **677**^8^	^1^OH(*v*);^2^CH(*v*);^3^NH(v);^4^NH(*δ,β);* ^5^CH(*δ,β);*^6^CO_3_^2−^;^7^CO(*v*);^8^COO-(*v*_*sym*_); ^9^COO-(*v*_*asym*_);^10^COO;^11^OH(*δ,β);*^12^Si-O; ^13^(CO₃^2^⁻)(*v*_*sym*_); ^14^CO_3_^2−^ (*β*)	490, 1400, 1515, 1645,1670, 1690
	Parchment	3582^1^, 3529^3^, 3417^3^, *3361*^2,3^, 3276^2^, ***2937***^2^, **2564**^6^, *2464*^7^, *2256*^2^, **1972**^6^, *1662*^1,4,11^, ***1556***^4,9^, **1467**^10^, *1354*^5,6^, **1051**^13^, **809**^14^, **742**^8^, **680**^8^	^1^OH(*v*);^2^CH(*v*);^3^NH(v);^4^NH(*δ,β);* ^5^CH(*δ,β);*^6^CO_3_^2−^;^7^CO(*v*);^8^COO-(*v*_*sym*_); ^9^COO-(*v*_*asym*_);^10^COO;^11^OH(*δ,β);* ^12^Si-O;^13^(CO₃^2^⁻)(*v*_*sym*_); ^14^CO_3_^2−^ (*β*)	490, 1385, 1500, 1645,1665, 1690
**VG + LW + GA**	Paper	*3981*^1^, 3510^3^, *3323*^2,3^, ***2975***^2^, *2463*^5^, *2136*^2^, *1660*^1,9^, ***1639***^1,6,9^, ***1597***^7^, ***1475***^8^, ***1166***^10,11^, **1098**^10,11^, **817**^13^, **792**^14^, **708**^12,13^**, 682**^8^	^1^OH(*v*);^2^CH(*v*);^3^CH(*δ,β);*^4^CO_3_^2−^; ^5^CO(*v*);^6^COO-(*v*_*sym*_);^7^COO(*v*_*asym*_); ^8^COO;^9^OH(*δ,β);*^10^Si-O;^11^(CO₃^2^⁻)(*v*_*sym*_); ^12^CO_3_^2−^(*β*);^13^Pb-O;^14^Pb-OH(*β*)	510, 1330, 1380, 1520, 1650
	Parchment	*3550*^1,3^, *3427*^3^, *3313*^2,3^, *2930*^2^, **2697**^6^, **2568**^6^, *2051*^2^, **1969**^6^, *1645*^1,4,11^, ***1608***^1,4,8,11^, ***1501***^9^, ***1473***^10^, ***1356***^5,6^, ***1158***^12,13^, **811**^15^, **790**^16^, **752**^14,15^, **699**^8,14,15^, **674**^10^	^1^OH(*v*);^2^CH(*v*);^3^NH(v);^4^NH(*δ,β);* ^5^CH(*δ,β);*^6^CO_3_^2−^;^7^CO(*v*);^8^COO-(*v*_*sym*_); ^9^COO-(*v*_*asym*_);^10^COO;^11^OH(*δ,β);* ^12^Si-O;^13^(CO₃^2^⁻)(*v*_*sym*_);^14^CO_3_^2−^(*β*); ^15^Pb-O; ^16^Pb-OH(*β*)	510, 1145, 1310, 1370,1550, 1650
**VG + LW + EG**	Paper	*3536*^1,3^, *3293*^2,3^, *2930*^2^, *2464*^5^, **1728**^8,13^, *1668*^1,4,8,11^, ***1644***^1,4,8,11^, ***1555***^9,13^, ***1502***^9^, ***1360***^5,6^, **1101**^12,13^, **1047**^12,13^, **836**^15^, **736**^14,5^, **705**^14,15^, **689**^10^	^1^OH(*v*);^2^CH(*v*);^3^NH(v);^4^NH(*δ,β);* ^5^CH(*δ,β);*^6^CO_3_^2−^;^7^CO(*v*);^8^COO-(*v*_*sym*_); ^9^COO-(*v*_*asym*_);^10^COO;^11^OH(*δ,β);*^12^Si-O; ^13^(CO₃^2^⁻)(*v*_*sym*_);^14^CO_3_^2−^(*β*);^15^Pb-O; ^16^Pb-OH(*β*)	495, 1335, 1385, 1520,1650, 1665
	Parchment	*3536*^1,3^, *3291*^2,3^, *2947*^2^, **2464**^5^, *2147*^2^, **1968**^6^, **1728**^8,13^, *1662*^4,8,11^, ***1644***^1,4,8,11^, ***1551***^9,13^, ***1502***^9^, **1476**^12,13^, ***1360***^5,6^, **1102**^12,13^, **1047**^12,13^, **836**^15^, **737**^15^, **706**^14,15^, **679**^10^	^1^OH(*v*);^2^CH(*v*);^3^NH(v);^4^NH(*δ,β);* ^5^CH(*δ,β);*^6^CO_3_^2−^;^7^CO(*v*);^8^COO-(*v*_*sym*_); ^9^COO-(*v*_*asym*_);^10^COO;^11^OH(*δ,β);* ^12^Si-O;^13^(CO₃^2^⁻)(*v*_*sym*_);^14^CO_3_^2−^(*β*); ^15^Pb-O; ^16^Pb-OH(*β*)	490, 1330, 1380, 1650
**CIN + LTY + LW + GA**	Paper	*3526*^1^, *3470*^2^, *3373*^2^, *3115*^2^, *2902*^2^, *2842*^2^, *2124*^2^, **1727**^8,12^, *1621*^1,10^, ***1595***^1,4,10^, ***1464***^4,8,11^, ***1130***^11,12^, **1103**^11,12^, **991**^5,11^, **885**^9,15^, **791**^15^, **710**^14^, **661**^8,11^	^1^OH(*v*);^2^CH(*v*);^3^CH(*δ,β);*^4^CO_3_^2−^; ^5^CO(*v*);^6^SO_2_(*V*_*asym*_);^7^SO_2_;^8^Pb-O; ^9^CO_3_^2−^(*β*);^10^OH(*δ,β);*^11^Si-O; ^12^CO_3_^2−^(*v*_*sym*_);^13^CO_3_^2−^(*v*_*asym*_); ^14^CO_3_^2−^ (*δ*);^15^*β*(Pb-OH)	615, 940, 1125, 1310,1520, 1660
	Parchment	3576^1,3^, *3392*^2,3^, *2909*^2^, 2670^6^, *2145*^2^, ***1725***^8,14^, *1606*^4,12^, ***1476***^6,10,13^, **1115**^13,14^, **958**^10,13^, **812**^10,11^, **747**^10,13^, **704**^10,11^, **684**^10,13,16^	^1^OH(*v*);^2^CH(*v*);^3^NH(v);^4^NH(*δ,β);* ^5^CH(*δ,β);*^6^CO_3_^2−^;^7^CO(*v*); ^8^SO_2_(*V*_*asym*_); ^9^SO_2_;^10^Pb-O;^11^CO_3_^2−^(*β*);^12^OH(*δ,β);* ^13^Si-O;^14^CO_3_^2−^(*v*_*sym*_);^15^CO_3_^2−^(*v*_*asym*_); ^16^CO_3_^2−^ (*δ*); ^17^*β*(Pb-OH)	615, 940, 1110, 1290,1545, 1650
**CIN + LTY + LW + EG**	Paper	*3529*^1,3^, *3297*^2,3^, *2920*^2^, *2854*^2^, **2410**^5^, *2124*^2^, *1647*^1,4,12^, ***1535***^4,6^, **1450**^6,10,13^, ***1386***^5,6^, **1245**^6,7,8^, **1152**^13,14^, **1092**^13,14^, **1047**^14^, **897**^11,17^, **753**^10,13^, **730**^10^, **681**^10,13,16^, **653**^10,13^	^1^OH(*v*);^2^CH(*v*);^3^NH(v);^4^NH(*δ,β);* ^5^CH(*δ,β);*^6^CO_3_^2−^;^7^CO(*v*);^8^SO_2_(*V*_*asym*_); ^9^SO_2_;^10^Pb-O;^11^CO_3_^2−^(*β*);^12^OH(*δ,β);* ^13^Si-O;^14^CO_3_^2−^(*v*_*sym*_);^15^CO_3_^2−^(*v*_*asym*_); ^16^CO_3_^2−^ (*δ*); ^17^*β*(Pb-OH)	630, 990, 1290, 1370, 1650
	Parchment	3534^1^, *3373*^2,3^, *3286*^2,3^, *2956*^2^, *2845*^2^, **2407**^5^, **1728**^8,14^, *1660*^4,12^, ***1544***^4,6^, ***1508***^4,6^, **1468**^15^, ***1350***^5,6^, **1103**^13,14^, **1046**^14^, **847**^11,17^, **771**^17^, **737**^10,13^, **702**^10,11^	^1^OH(*v*);^2^CH(*v*);^3^NH(v);^4^NH(*δ,β);* ^5^CH(*δ,β);*^6^CO_3_^2−^;^7^CO(*v*);^8^Pb-O; ^8^SO_2_(*V*_*asym*_);^9^SO_2_;^10^Pb-O;^11^CO_3_^2−^(*β*); ^12^OH(*δ,β);*^13^Si-O;^14^CO_3_^2−^(*v*_*sym*_); ^15^CO_3_^2−^(*v*_*asym*_);^16^CO_3_^2−^(*δ*); ^17^*β*(Pb-OH)	630, 990, 1290, 1370, 1650

DRIFTS spectral range: 4000–650 cm^−1^; HSI spectral range: 400–1700 nm. In **bold** = bands attributed to pigments; *cursive* = bands attributed to binders (gum Arabic, egg glair) and water (1640 cm^−1^); underlined = bands attributed to supports. Bands near 1086 cm^−1^ are associated with the calcite pigment used in the degreasing process [11]. Specific assignments are described in the body of the text. See Table 1 for authors’ names abbreviations

**Fig. 7:**
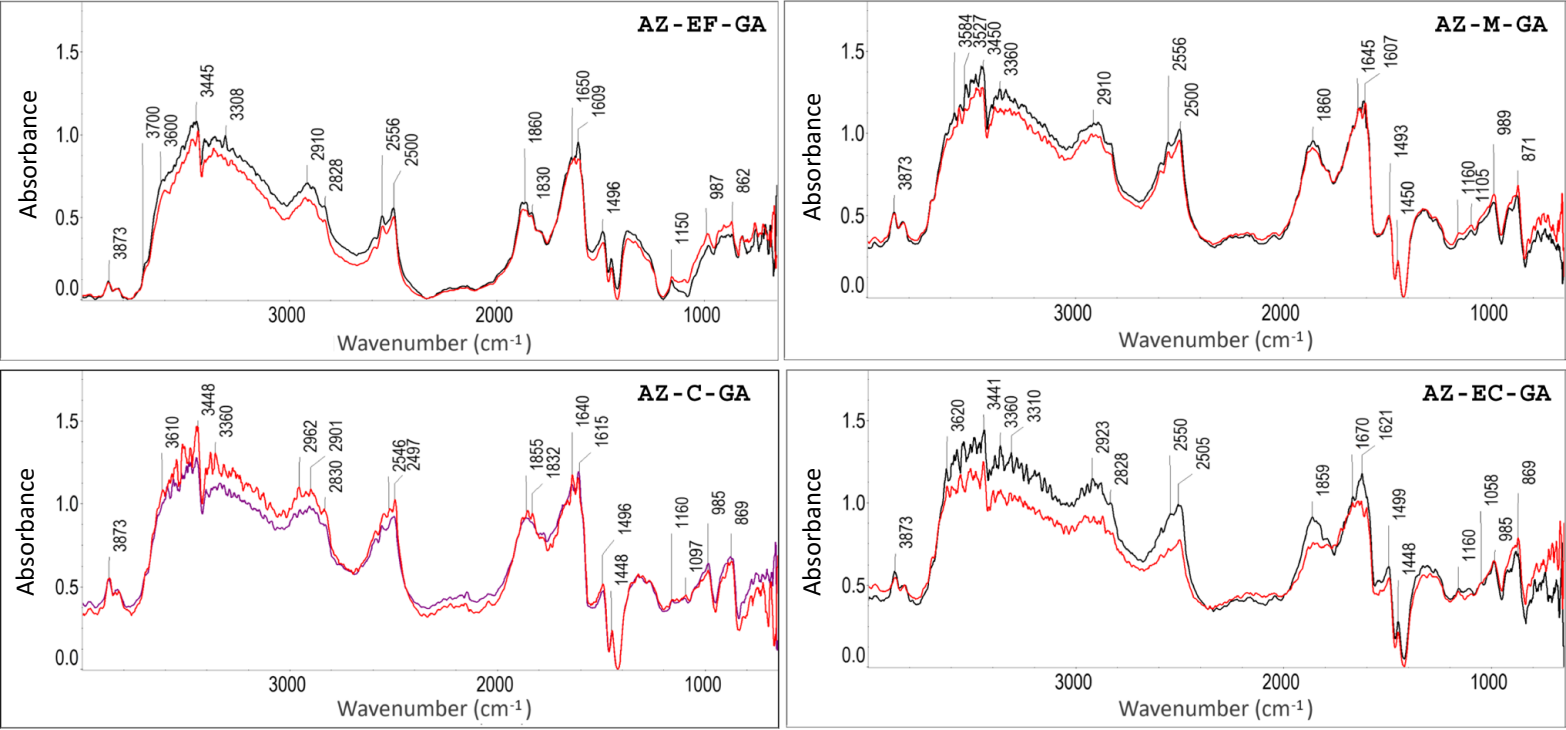
DRIFTS spectra of azurite (AZ) pigments mixed with gum Arabic (GA). Black, paper; red, parchment support. See Table 1 for authors’ name abbreviations

Spectral variations due to different particle sizes in azurite pigments were additionally studied with DRIFTS and HSI, since their influence was noticed in previous studies [36, 37]. Results of azurite-based mock-ups bound with gum Arabic on paper and parchment are presented in Fig. 7 and in Table 5. Band assignments for mock-ups bound with egg glair are not shown but are available in the Supplementary Information.

Azurite pigments exhibit typical bands associated with the asymmetric stretching of CO_3_^2^⁻ at 1496 and 1448 cm⁻^1^ [14], as well as bending at 862 cm⁻^1^ (δ of CO_3_^2^⁻) [47]. Bands close to 2500 cm⁻^1^ (*β* of CO_3_^2^⁻) have been described as an indicator of azurite by Vetter et al. (2019) [48]. Bands between 3600 and 3700 cm⁻^1^ and around 1150 cm⁻^1^ are attributed to OH(*v*) and CO(*v*) bonds originating from gum Arabic, while intense bands between 1640 and 1607 cm⁻^1^ are associated with vibrational movements of water molecules. DRIFTS analysis revealed spectral variations due to particle size, with coarser pigments (AZ-M, AZ-C, AZ-EC; see identified particle sizes in Table 3) showing higher intensities when compared to AZ-EF. Minor shifts in the bands were observed when placed on different supports, with these shifts being more pronounced on parchment supports. The main variations were found in the spectral range from 1690 to 1550 cm⁻^1^ (C = O and NH bonds).

**Table 5:** Main DRIFTS bands for azurite (different particle size) mock-ups mixed with gum Arabic on diverse supports

Mock-up	DRIFTS wavenumbers (cm^−1^)	DRIFTS assignments
*AZ-EF-GA on paper*	**3870*vw***^1^, **3823***vw*^1^, **3552*s***^**1**^, **3510*s***^**1**^, 3445*vs*^1,2^, **3399*s***^2^, 3153*s*^1^, 2909*m*^2^, **2550*m***^**3**^, **2497*m***^3^, **2140*vw***^2^, 1858*m*^2,4^, **1831*m***^2,4^, 1640*s*^1^, 1607*vs*^1,4^, **1488*m***^*4*^, **1446*w***^4^, 1366*w*^7^, **1155*w***^6^, **977*w***^6,9^, **865*m***^3,8^, **748*m***^***7***^, **704*m***^7^, **679*m***^5^	^1^OH(*v*); ^2^CH(*v*), ^3^CO_3_^2−^(*β*); ^4^CO_3_^2−^ (*v*_*asym*_); ^5^Si-O(*v*); ^6^CO(v); ^7^CH(*δ,β)*; ^8^CO_3_^2−^ (*δ*); ^9^OH(*δ, β)*
*AZ-EF-GA on parchment*	**3871***vw*^1^, **3827*vw***^1^, **3557*s***^1^, 3438*vs*^1,2,13^, 3362*s*^2,13^, 2922*m*^2^, 2549*m*^3^, 2495*m*^3^, **2140*vw***^2^, 1871*m*^2,4^, **1832 m**^2,4^, 1624*s*^1,4,12^, 1607*vs*^1,4,12^, **1485*m***^4^, **1446*w***^4^, 1365*w*^7^, **1153w**^6^, 1096*vw*^*10*^, **983*m***^6,9^, 904*m*^11^, **866*m***^3,8^, **753*m***^7^, **707*m***^7^, **682*m***^5^, 666*m*^11^	^1^OH(*v*); ^2^CH(*v*), ^3^CO_3_^2−^(*β*); ^4^CO_3_^2−^ (*v*_*asym*_); ^5^Si-O(*v*); ^6^CO(v); ^7^CH(*δ,β)*; ^8^CO_3_^2−^(*δ*); ^9^OH(*δ, β);* ^10^CO_3_^2−^ (*v*_*sym*_); ^11^Si-O(*V*_*as*_); ^12^NH(*δ,β*); ^13^NH(*v*)
*AZ-M-GA on paper*	**3870*m***^1^, **3828*m***^1^, **3500*vs***^1^, 3450*vs*^1^, 3401*vs*^1,2^, 3146*vs*^1^, 2888*vs*^2^, **2550*s***^3^, 2154*w*^2^, **1853*s***^**2**,4^, 1628*s*^1,4^, 1610*vs*^1,4^, **1489*m***^4^, **1446*w***^4^, 1313*m*^7^, **1264*m***^11^, 1095 m^10^, 986*m*, **875*m***, 775*m*, **739*w****, 697w, 678w*	^1^OH(*v*); ^2^CH(*v*), ^3^CO_3_^2−^(*β*); ^4^CO_3_^2−^ (*v*_*asym*_); ^5^Si-O(*v*); ^6^CO(v); ^7^CH(*δ,β)*; ^8^CO_3_^2−^(*δ*); ^9^OH(*δ, β);* ^10^CO_3_^2−^ (*v*_*sym*_); ^11^Si-O(*V*_*as*_)
*AZ-M-GA on parchment*	**3870*m***^1^, **3825*m***^1^, **3557*vs***^1^, 3443*vs*^1,2,13^, **3313*vs***^2,13^, **2920*s***^2^, **2550*s***^3^, **2499*s***^3^, 2179*w*^2^, **1857*s***^**2**,4^, 1637*vs*^1,12^, 1603*vs*^1,4,12^, **1490*m***^4^, **1446*w***^4^, 1326*m*^7^, 1093*m*^*10*^, **985*m***^6,9^, **870*m***^3,8^, **746*m***^7^, **675*m***^5^	^1^OH(*v*); ^2^CH(*v*), ^3^CO_3_^2−^(*β*); ^4^CO_3_^2−^ (*v*_*asym*_); ^5^Si-O(*v*); ^6^CO(v); ^7^CH(*δ,β)*; ^8^CO_3_^2−^(*δ*); ^9^OH(*δ, β)¸* ^10^CO_3_^2−^ (*v*_*sym*_); ^12^NH(*δ,β*); ^13^NH(*v*)
*AZ-C-GA on paper*	**3867*m***^1^, **3822*m***^1^, **3553*vs***^1^**, 3506*vs***^1^, 3402*vs*^1,2^, 2954*vs*^2^, 2832*s*^2^, **2492*vs***^3^, 2152*m*^2^, **1853*s***^**2**,4^, 1829*s*^2,4^**,** 1670*vs*^1^, 1637*vs*^1^, 1611*vs*^1,4^, **1491*m***^4^, **1446*w***^4^, 1319*m*^7^, 1090*m*^*10*^, **984*m***^6,9^, **868*m***^3,8^, 775*w*^7^, 746*m*^7^, **680*m***^5^	^1^OH(*v*); ^2^CH(*v*), ^3^CO_3_^2−^(*β*); ^4^CO_3_^2−^ (*v*_*asym*_); ^5^Si-O(*v*); ^6^CO(v); ^7^CH(*δ,β)*; ^8^CO_3_^2−^(*δ*); ^9^OH(*δ, β);* ^10^CO_3_^2−^ (*v*_*sym*_)
*AZ-C-GA on parchment*	**3871*m***^1^, **3828*m***^1^, 3589*vs*^1^, **3547*vs***^1^**,** 3496*vs*^1,2,13^, 3402*vs*^1,2,13^, 3119*s*^2^, **2904*s***^2^**,** 2554 s^3^, **2497*s***^3^**, 2145*m*, 1834*s***, 1639*vs*^1,12^, 1609*vs*^1,4,12^, 1**489*m***^4^, **1447*w***^4^, 1323*m*^7^, 1096*w*^*10*^**, 983*m***^6,9^, **875*m***^3,8^, **753*m***^7^, **700*m***^7^, **677*m***^5^	^1^OH(*v*); ^2^CH(*v*), ^3^CO_3_^2−^(*β*); ^4^CO_3_^2−^ (*v*_*asym*_); ^5^Si-O(*v*); ^6^CO(v); ^7^CH(*δ,β)*; ^8^CO_3_^2−^(*δ*); ^9^OH(*δ, β);* ^10^CO_3_^2−^ (*v*_*sym*_); ^12^NH (*δ,β*); ^13^NH(*v*)
*AZ-EC-GA on paper*	**3871*w***^1^, 3835*w*^1^, 3622*vs*^1^, 3575*vs*^1^, **3553*vs***^**1**^, 3439*vs*^1,2^, 3399*vs*^1,2^, 3195*vs*^1^, 2942*vs*^2^, **2506*s***^3^, 2159*m*^2^, **1860*s***^**2**,4^, 1620*vs*^1,4^, **1493*m***^4^, **1446*w***^4^, 1303*m*^7^, 1103*m*^*10*^**, 985*m***^6,9^, **880*m***^**3**,8^, 788*m*^7^, 738*m*^7^, **697*m***^7^**,** 678*s*^5^	^1^OH(*v*); ^2^CH(*v*), ^3^CO_3_^2−^(*β*); ^4^CO_3_^2−^ (*v*_*asym*_); ^5^Si-O(*v*); ^6^CO(v); ^7^CH(*δ,β)*; ^8^CO_3_^2−^(*δ*); ^9^OH(*δ, β);* ^10^CO_3_^2−^ (*v*_*sym*_)
*AZ-EC-GA on parchment*	**3868*m***^1^, 3824*m*^1^, 3596*vs*^1^, **3553*vs***^1^, **3506*vs***^1^, 3443*vs*^1,2^, 3393*vs*^1,2^, 2956*s*^2^, **2497*s***^3^, **1835*s***^**2**,4^, 1665vs^1,12^, 1624*vs*^1,4^, 1598*s*^1,12^*,* **1491*m***^4^, **1447*w***^4^, 1292*m*^7^, 1157*m*^10^, **986*m***^6,9^, **869*m***^3,8^, 747*m*^7^, **704*m***^7^, 666*s*^11^	^1^OH(*v*); ^2^CH(*v*), ^3^CO_3_^2−^(*β*); ^4^CO_3_^2−^ (*v*_*asym*_); ^5^Si-O(*v*); ^6^CO(v); ^7^CH(*δ,β)*; ^8^CO_3_^2−^ (*δ*); ^9^OH(*δ, β;* ^10^CO_3_^2−^ (*v*_*sym*_); ^11^Si-O(*V*_*as*_); ^12^NH (*δ,β*); ^13^NH(*v*)

Relative intensities: *vs* very strong, *s* strong, *m* medium, *w* weak, *vw* very weak. *ν* = *stretching*; *ν*_*asym*_ = antisymmetric stretching; *ν*_*sim*_ = symmetric stretching; *δ* = bending (*scissoring*); *β* = bending (*wagging/twisting*). Band numbers marked in bold font correspond to those identified for azurite in previous studies [14, 24, 44, 47, 51, 56–58]

**Fig. 8:**
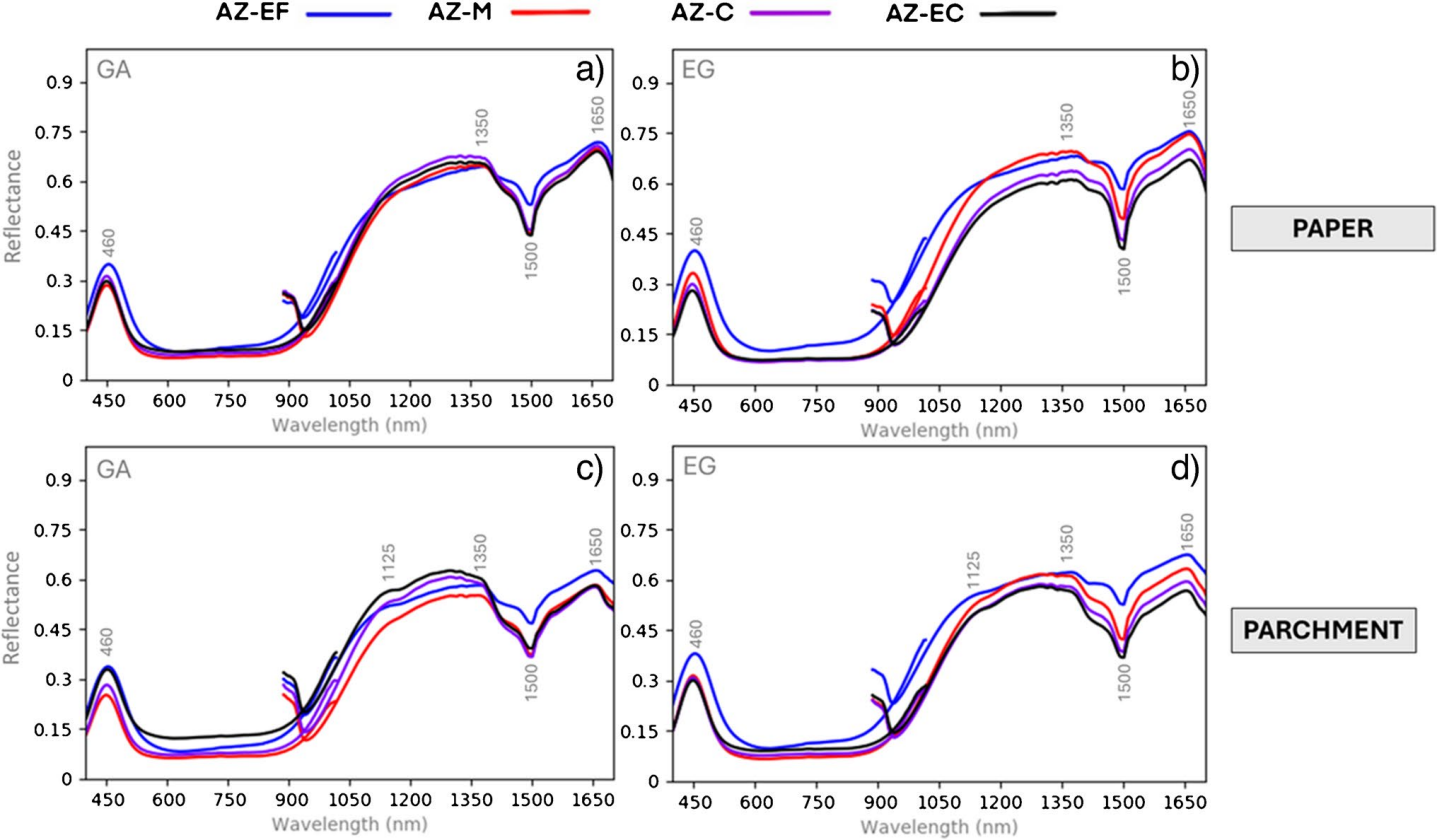
Reflectance spectra of azurite-based mock-ups with different particle sizes and binders. GA = gum Arabic; EG = egg glair. See Table 1 for authors’ name abbreviations

Additionally, the influence of particle size is discussed using HSI reflectance spectral data (Fig. 8). Here, main variations are observed in reflectance intensity, which decreases proportionally to particle size in pigments bound with gum Arabic. However, a slightly different behavior is observed for AZ-EF-based samples, which may be attributed to a stronger pigment-binder interaction, as previously noted by Cardell et al. (2017) [37]. In contrast, pigments bound with egg glair exhibit an inverse pattern, with the fine grain mock-up showing the highest reflectance intensity, while the coarse grain sample has the lowest. These results highlight the combined influence of pigment particle size and binder type on reflectance spectra.

#### Illuminated manuscripts

**Fig. 9:**
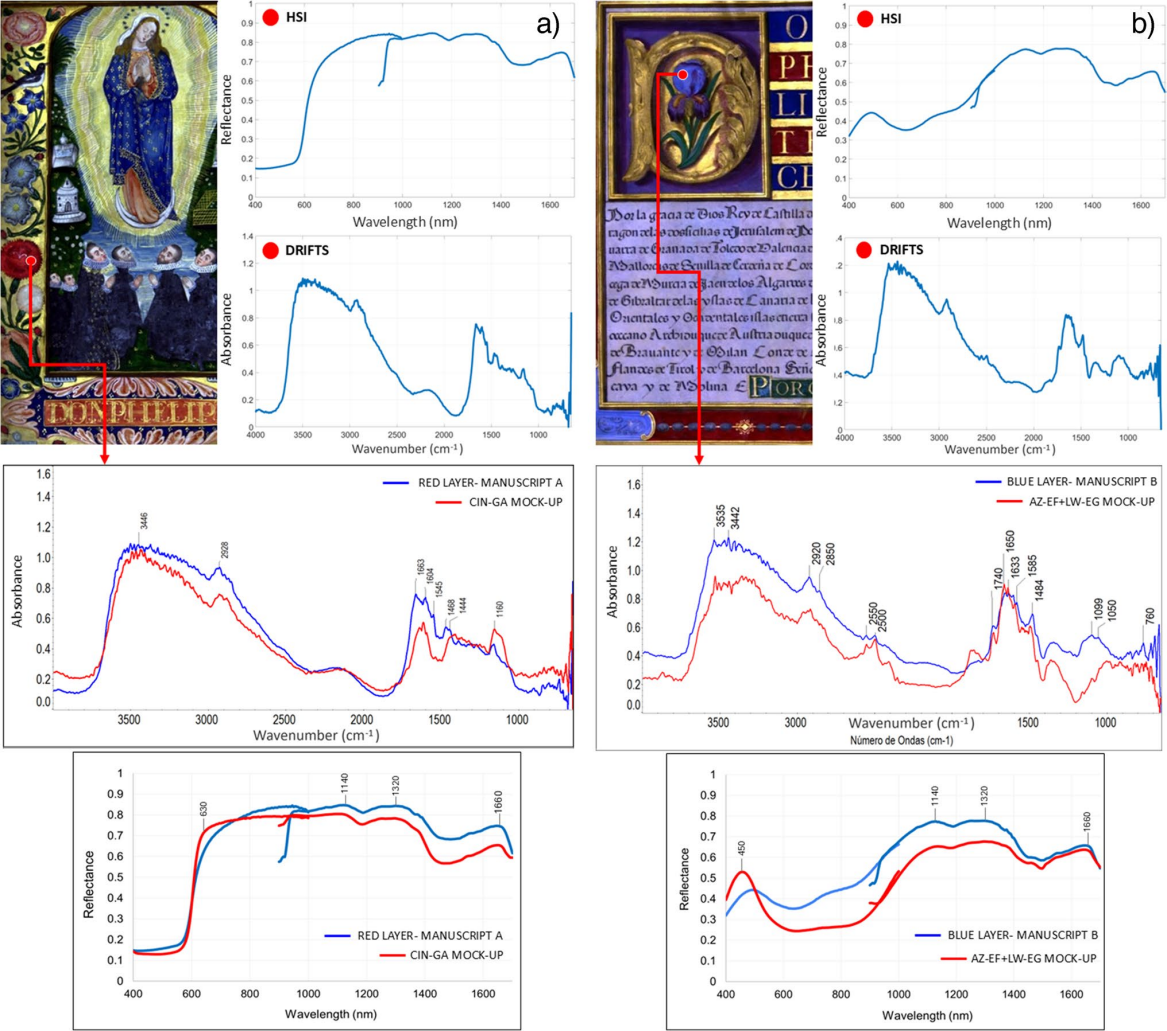
Non-invasive HSI and DRIFTS analysis of manuscripts **a** A and **b** B, and spectral comparison between historical manuscripts (blue) and database spectra (red) of mock-ups CIN + GA on parchment and AZ-EF + LW + EG on parchment

Finally, two manuscripts from the Archive of the Royal Chancellery are presented to exemplify the applicability of the database for material analyses in historical documents. Here, manual spectral comparison was used to identify possible binders and pigments or dyes. In the red painting layers of manuscript A (sixteenth century, parchment), previous analyses using X-ray fluorescence (XRF) suggested the presence of mercury (Hg). DRIFTS and HSI results of the measured spot (Fig. 9a) confirmed the presence of cinnabar (see Tables 1 and 4), which was most likely bound with gum Arabic, after comparison with the mock-ups of our database. In the blue pictorial layer of manuscript B (seventeenth century, parchment), DRIFTS results indicate a homogeneous mixture of azurite (AZ-EF) and lead white (LW) (Fig. 9b). Comparison between AZ-EF + LW spectra mixed with either gum Arabic or egg glair suggested the second as the used binder. HSI reflectance values of both illuminated manuscripts also correspond with those of the captured CIN-GA and AZ-EF + LW-EG mock-ups, which accentuates the combined value of both analytical techniques. The number of variables included in the database highlights its novelty, since different aspects, such as type of pigment, dye, binder, and support, or the type of layer (mixture, superimposed layers) can be resolved in decorated manuscripts.

### Color analysis

**Fig. 10:**
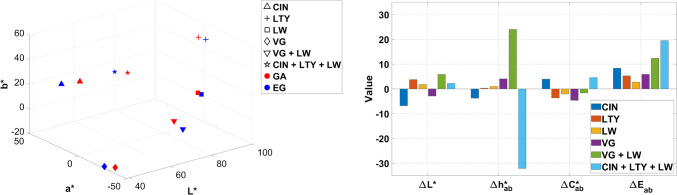
Effect of the binder on the color of paint mock-ups. Chromaticity coordinates and color difference values considering GA (gum Arabic) as reference for the two binders on paper substrate for different mock-ups. L* (lightness); a* and b* (chromatic coordinates); C*_ab_ (chroma); h*_ab_ (hue); ΔL* (lightness/darkness); ΔC*_ab_ (chroma difference); Δh*_ab_ (hue difference); ΔE*_ab_ (total color difference). See Table 1 for authors’ name abbreviations

**Fig. 11:**
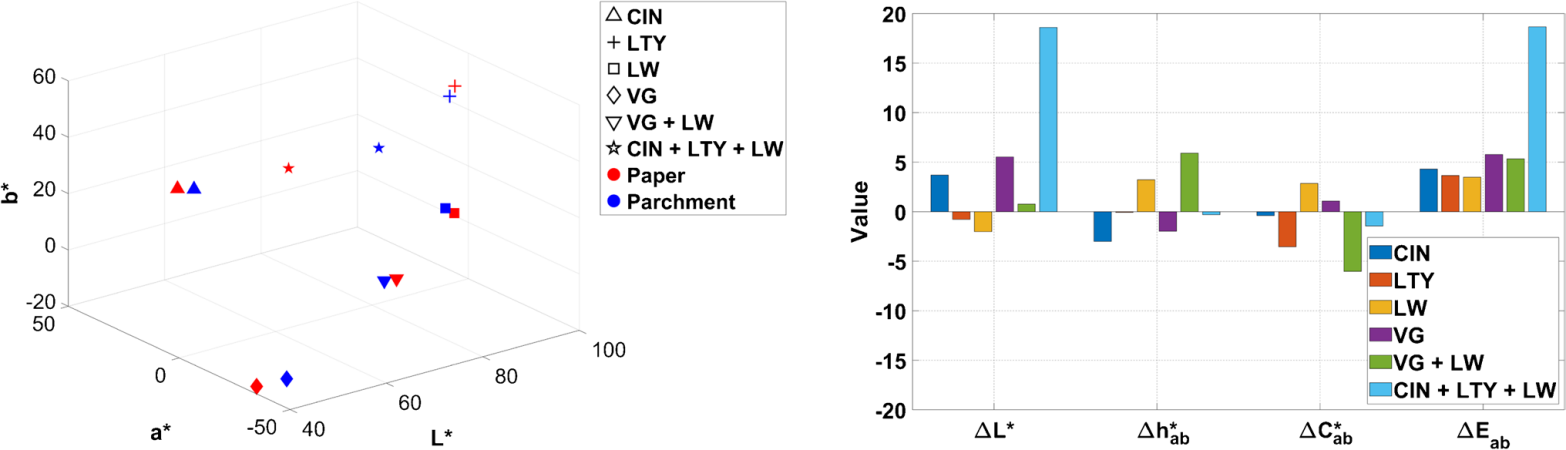
Effect of the support on the color of paint mock-ups. Chromaticity coordinates and color difference values considering paper as reference for samples containing GA (gum Arabic) and deposited on paper or parchment. L* (lightness); a* and b* (chromaticity coordinates); C*_ab_ (chroma); h*_ab_ (hue); ΔL* (lightness/darkness); ΔC*_ab_ (chroma difference); Δh*_ab_ (hue difference); ΔE*_ab_ (total color difference). See Table 1 for name abbreviations

**Fig. 12:**
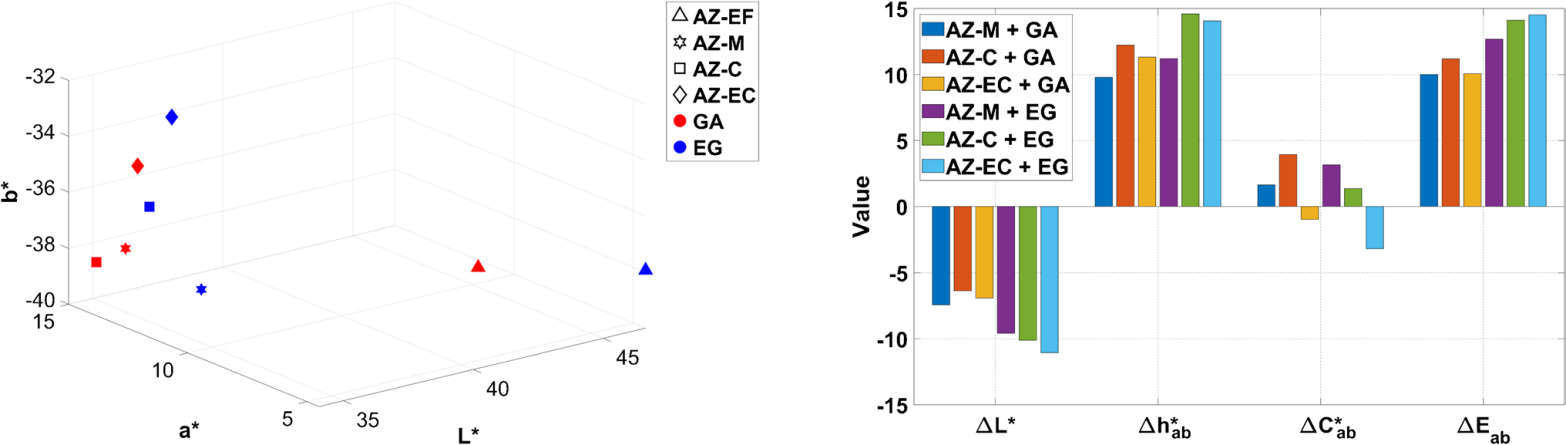
Chromaticity coordinates and color difference considering the mock-up containing the finest azurite pigment (AZ-EF) as reference for mock-ups made of azurite with different particle sizes (AZ-M, AZ-C, AZ-EC). L* (lightness); a* and b* (chromatic coordinates); C*_ab_ (chroma); h*_ab_ (hue); ΔL* (lightness/darkness); ΔC*_ab_ (chroma difference); Δh*_ab_ (hue difference); ΔE*_ab_ (total color difference). See Table 1 for name abbreviations

In this section, the results of the color characterization of the mock-ups are discussed, considering three different variables: the effect of binders (Fig. 10), supports (Fig. 11), and particle size for azurite pigments (Fig. 12). In this study, only ΔE*_ab_ color differences are presented, although CIEDE00 color differences [37] for all mock-ups can be accessed through the Supplementary Information (*Chromatic_Coordinates_HSI_Hyperdoc*).

The impact of the binder is shown in Fig. 10 for some selected examples of biphasic, triphasic, and quadriphasic mixtures included in the database. In approximately 80% of the mock-ups, lightness (L*) is higher in mixtures bound with egg glair than in those containing gum Arabic, with higher differences observed in samples containing lead white (LW). This can be attributed to the higher transparency of painting layers bound with gum Arabic when compared to those bound with egg glair, where the influence of the support on color is greater. Slight increases and decreases in chroma (C*_ab_) and hue (h*_ab_) were observed equally when comparing both binders, suggesting that there is no substantial difference in these variables. However, higher hue variations occurred in the triphasic (for example, VG + LW + binder) and quadriphasic mixtures (CIN + LTY + LW + binder), indicating that multicomponent mock-ups are more sensitive to binder changes, while simpler mixtures (LW and LTY biphasic mixtures in Fig. 10) are less influenced. The highest color difference (ΔE*_ab_) also corresponds to the quadriphasic mock-ups (ΔE*_ab_ = 19.50 in paper and ΔE*_ab_ = 25.68 in parchment), corroborating the larger impact of the binder on color in complex mixtures.

The influence of the support on the color of pigment mixtures was also studied (Fig. 11). L* values are higher for parchment substrates in 56% of the samples, with the greatest difference observed in the quadriphasic mixture composed of CIN, LTY, and LW bound with GA (ΔL* = 18.60). The average lightness of the parchment samples is 67.52, compared to 67.46 for the paper samples, indicating minimal differences for this variable. However, this small difference may be attributed to the effect of light reflected by the substrate contour surrounding the painted area, with parchment being more reflective than paper.

Coordinates a* and b* shift depending on the pigment combination, although a* values are generally higher in mock-ups on paper, while b* values increase on parchment supports, as expected based on the intrinsic properties of the material. Chroma and hue differences (ΔC*_ab_ and Δh*_ab_) are generally minor, with higher values of both in 64% of the mock-ups placed on paper. On average, the influence of the support on color tends to be lower than the influence of the binder, suggesting that there is generally sufficient covering of the support.

Regarding the influence of pigment particle size on the color of painting mock-ups made with azurite (AZ) (Fig. 12), results show slight increases in L* values with decreasing particle size for both binders and supports. This effect is most noticeable in samples containing extra-fine azurite (AZ-EF). HSI spectra shown in Fig. 8 confirm a trend toward greener hues for finer-grain azurite pigments. This, along with the lower a* values observed in Fig. 12a, aligns with findings in previous studies [36], which mention that azurite pigments with smaller particle sizes tend to exhibit higher reflectance and L* values. Additionally, according to the obtained b* values, the pigment AZ-M bound with GA and pigment AZ-C bound with EG are the bluest mock-ups. As expected, higher color differences are observed for coarser pigments (e.g., AZ-C, AZ-EC) compared to medium grain size (e.g., AZ-M) and extra-fine azurite pigment (AZ-EF). These differences are more pronounced in mock-ups mixed with EG (Fig. 12b).

Finally, the study of the influence of paint application mode on the color (Fig. 1) revealed higher luminosity (L*) and lower chroma (C*_ab_) values when compared to biphasic mixtures (e.g., VG + binder). This is primarily due to the addition of lead white pigment, whether mixed homogeneously in the painting mixture or deposited as the upper layer on the surface of the mock-ups. In general, superimposed mixtures exhibit higher lightness when lead white is added, while chroma (C*_ab_) is reduced. Instead of combining the color contributions of both components, the lead white pigment dominates the color. Although lead white’s covering power is typically high, its luminosity is influenced by the binder used, with mock-ups bound with egg glair showing higher luminosity compared to those bound with gum Arabic. Additionally, higher L* values can be attributed to greater similarities in luminosity when compared to the support. In all cases, the highest L* values were observed when exclusively selecting the white superimposed layer as the region of interest (ROI). This highlights possible variations in obtained values when analyzing historical documents, where the boundaries between areas of different colors may not be as clearly defined as in laboratory-produced mock-ups

In summary, the factor that most affects the final color of the sample is the type of binder, resulting in an average color difference of ΔE*_ab_ = 9.62. In the context of this study, 92% of the color differences exceed 3, meaning they are visually perceptible [38]. Regarding the influence of supports, 64% of the color differences are perceptible, with an average ΔE*_ab_ = 4.91. Finally, for azurite particle size, no clear trend is observed, with the bluest samples being those with intermediate particle sizes (M and C), which exhibit partially coincident size ranges.

## Concluding remarks and future perspectives

This study presents a comprehensive spectral library generated by complementing DRIFTS and HSI data from 156 painting mock-ups prepared using historical recipes. The mock-ups include biphasic, triphasic, and quadriphasic mixtures on diverse supports, created to identify materials in historical documents.

HSI has proven invaluable in complementing DRIFTS data, providing both reflectance spectra and chromatic coordinates, which offer objective and systematic data on color and surface appearance. Portable DRIFTS has shown to be effective for artwork analysis, as comparisons between contact and non-contact measurements presented minimal variations. However, some limitations, such as interference from supports leading to erroneous interpretations and the inability to detect trace materials in non-pure pigments or thin layers, need to be addressed. Additionally, the selection of optimal spectral ranges is critical when analyzing pigments with characteristic bands below the spectral measurement range, such as lead, mercury, or arsenic-based pigments (e.g., minium, cinnabar, orpiment) or pigments comprised of iron oxides (e.g., hematite). At present, portable DRIFTS is considered more suitable for the analysis of supports, binders, and certain pigments such as carbonates (azurite, malachite, lead white, and calcite), silicates (lapislazuli), glass pigments (blue smalt), and acetates (verdigris). Future research will incorporate ATR-FTIR to address these gaps and provide comparative results with the data presented here.

Surface irregularity, particularly on parchment, posed challenges in obtaining high-quality DRIFTS and HSI data. These issues were mitigated by extending acquisition times for DRIFTS and adding weights to flatten the mock-ups for HSI. However, ensuring flatness remains a significant challenge for historical documents due to their sensitivity to manipulation and environmental fluctuations. Determining the composition and contribution of each painting component and their interactions is crucial, but limited information exists in the literature. The presented database, which includes multiple variables (support type, binder type, pigment particle size, paint application method, and contact/non-contact measurements), serves as an innovative tool for pigment and dye characterization. Although results refer to illuminated documents, the database presents a broader applicability for different types of artworks, given the set of included pure pigments’ DRIFTS spectra. Additionally, the potential applicability of extending the obtained data to other polychrome surfaces where either gum Arabic or egg glair are used as binders constitutes one of the future aims of this research.

Additionally, methods for automated material identification using HSI and DRIFTS data are being explored. The database includes 400 reflectance spectra per mock-up, which can be used to train machine learning models. Ongoing research aims to develop spectral unmixing methods to identify and quantify individual pigments and dyes in historical painting mixtures using non-invasive techniques.

## Data Availability

The database is publicly available online on Figshare [59]: https://doi.org/10.6084/m9.figshare.28639103.v1
